# Protective Effect on Bone of Nacre Supplementation in Ovariectomized Rats

**DOI:** 10.1002/jbm4.10655

**Published:** 2022-07-15

**Authors:** Dung Kim Nguyen, Norbert Laroche, Arnaud Vanden‐Bossche, Marie‐Thérèse Linossier, Mireille Thomas, Sylvie Peyroche, Myriam Normand, Yacine Bertache‐Djenadi, Thierry Thomas, Hubert Marotte, Laurence Vico, Marie‐Hélène Lafage‐Proust, Marthe Rousseau

**Affiliations:** ^1^ U1059 SAINBIOSE, INSERM Jean Monnet University, University of Lyon, Mines Saint‐Étienne Saint‐Étienne France; ^2^ Department of Rheumatology Hôpital Nord, CHU Saint‐Etienne Saint‐Etienne France; ^3^ University of Lyon, INSA‐Lyon, CNRS, MATEIS (UMR 5510) Villeurbanne France

**Keywords:** BONE, NACRE, OSTEOPOROSIS, OVARIECTOMY, RATS

## Abstract

Nacre has emerged as a beneficial natural product for bone cells and tissues, but its effect was only studied by gavage in the ovariectomized mouse model. We sought to assess the antiosteoporotic effect of nacre through a nutritional supplementation in the ovariectomized rat model. Sixteen‐week‐old female Wistar rats were either Sham‐operated or bilateral ovariectomized (OVX) and then fed with standard diet (Sham and OVX groups) or standard diet supplemented with either 0.25% CaCO_3_ or nacre (OVX CaCO_3_ and OVX Nacre group, respectively) for 28 days (*n* = 10/group). The bone microarchitecture was assessed at appendicular and axial bones by micro‐computed tomography (μCT). Histomorphometric analysis was performed to determine cellular and dynamic bone parameters. Bone metabolism was also evaluated by biochemical markers and gene expression levels. Nacre‐based diet prevented the OVX‐induced bone loss better than that of the CaCO_3_ supplement, given the significant changes in trabecular bone volume fraction (BV/TV) both at the femoral distal metaphysis (difference, 35%; *p* = 0.004) and at the second lumbar spine (difference, 11%; *p* = 0.01). Trabecular osteoclast surfaces (Oc.S/BS) were also 1.5‐fold lower at the tibial proximal metaphysis in OVX Nacre group compared with OVX CaCO_3_ group (*p* = 0.02)_._ By principal component analysis (PCA), OVX Nacre group formed a cluster away from OVX group and with a trend closest to Sham group. These data were consistent with biological measurements demonstrating a positive profile related to nacre supplementation, which blunted an increase in serum CTX level and enhanced serum P1NP secretion 14 days post‐OVX compared with CaCO_3_ supplementation. *Bmp2* mRNA expression in OVX Nacre group was +1.76‐fold (*p* = 0.004) and +1.30‐fold (*p* = 0.20) compared with OVX and OVX CaCO_3_ groups, respectively. We conclude that supplementation with nacre could effectively limit bone loss induced by estrogen deficiency just after OVX in rats by modulating the negative imbalance of bone turnover. © 2022 The Authors. *JBMR Plus* published by Wiley Periodicals LLC on behalf of American Society for Bone and Mineral Research.

## 1 Introduction

Osteoporosis is a common and widespread skeletal disease that increases the risk of fragility fractures.^(^ ^1^ ^)^ It is characterized by an imbalance of bone remodeling that leads to a systemic impairment of bone mass, strength, and microarchitecture. Although both men and women gradually lose bone as part of the aging process, a greater loss is observed in postmenopausal women associated with estrogen deficiency.^(^ ^2^ ^)^ The incidence of fracture increases with age in both sexes, but age‐adjusted rates are 49% greater in females than in males.^(^ ^3^ ^)^ In addition, after an initial fracture, women increase their risk of sustaining a second fracture by 36%,^(^ ^4^ ^)^ and from the age of 60 years, the residual lifetime risk of fracture is ~50% in women.^(^ ^5^ ^)^ Thus, postmenopausal osteoporosis related to its clinical consequences, including morbidity and mortality, imposes a significant health care problem to the society.

Today, there is a need to develop novel approaches for osteoporosis treatment with fewer adverse effects than current therapies. It is encouraging that products from natural origin, such as plant‐derived extracts,^(^ ^6^, ^7^ ^)^ marine animal–derived products,^(^ ^8^, ^9^, ^10^ ^)^ and fungi's metabolite,^(^ ^11^ ^)^ appear to be reliable sources for the development of antiosteoporotic agents due to their efficacy and safety. Nacre (mother‐of‐pearl) has been considered one of the potential candidates.

Nacre is produced by mollusks and is composed of crystalline calcium carbonate (CaCO_3_) embedded in an organic matrix.^(^ ^12^ ^)^ This inorganic microstructure coupled with the organic phase contributes to the bioactivity of nacre as a novel biomaterial.^(^ ^13^ ^)^ Furthermore, several nacre matrix factors have been identified and known to have biological activities on bone cells. Protein PFMG1^(^ ^14^ ^)^ and water/ethanol‐soluble matrix^(^ ^15^, ^16^ ^)^ have been derived from *Pinctada*'s pearl oyster family, which accelerate osteoblast differentiation. Beside the regulation of mineralization, protein N16^(^ ^17^ ^)^ and other molecules^(^ ^18^ ^)^ from nacre have been demonstrated to inhibit osteoclastogenesis. These properties of nacre have directed research toward its effect on osteoporosis.

The preclinical studies revealed that nacreous factors prevent induced bone loss.^(^ ^8^, ^14^ ^)^ Most of these studies have been conducted in ovariectomized mouse model under oral administration (gavage) or virus injection techniques, whereas our experimental powders were integrated in dietary foods, which is considered as a classic nutritional supplementation in human.

In the large variety of animal species, including rodents, non‐rodents (ie, rabbits, dogs, primates) have been used as animal models in osteoporosis research, and no single animal species duplicates all the characteristics of human osteoporosis, and each species exhibits its own limitation.^(^ ^19^, ^20^ ^)^ Here, we performed the ovariectomy, which mimics a condition similar to menopause, and also chose rat model, which bears a strong resemblance to human osteopenia, both in its anatomical features as well as in the transitional and steady stages of the bone dynamics.^(^ ^19^ ^)^

Moreover, it is known that in osteoporotic women, the first and most severe bone changes occur in the spongy bone of the vertebral body, whereas in rats they predominantly involve the trabecular bone of the metaphyseal region of the long bones.^(^ ^21^ ^)^ Also, the bone sites display different responses to bone loss stimulus related to their structural and metabolic characteristics.^(^ ^6^, ^19^ ^)^ Hence, we thought that investigating the effect of nacre at both appendicular and axial bones would provide more translational insights of this novel research direction.

To test the hypothesis that dietary nacre powder may have a protective effect against bone loss in the ovariectomized rat, we pursue two specific aims: (i) to quantify the prevention of bone loss due to estrogen deficiency at several skeletal sites and (ii) to explore the nacre action mechanisms through biomarkers involved in bone metabolism.

## 2 Materials and Methods

### 2.1 Animal experimentation

Eight‐week‐old female Wistar rats (mean ± SD body weight 166.8 ± 11.8 g) were housed under controlled conditions (22°C ± 1°C, 50% to 80% relative humidity; 12‐hour light/dark cycle) in groups of two animals per cage with free access to water and food *ad libitum*. The animals benefited from quality attention and care by qualified personnel, both during and outside of procedures, to ensure optimal well‐being throughout the study. This study was carried out with the approval of the French Ministry of Research and Innovation (approval no. APAFIS # 13401‐2018020615412128 v6). Animal procedures were performed in an accredited animal facility (authorization no. 42180801) at the PLEXAN (Platform for Animal Experimentation and Analysis, Medical School, Saint‐Etienne, France) with the approval of the Animal Ethics Committee and in accordance with directive 2010/63/EU and the Principles of Laboratory Animal Care recommended by the National Society for Biomedical Research in France. All animal handling and experimental protocols followed the ethical standards established in the 1964 Declaration. Sixteen‐week‐old animals (mean ± SD body weight 237.6 ± 14.1 g) were assigned to one of the four groups (*n* = 10 per group): (i) Sham; (ii) OVX; (iii) OVX with CaCO_3_ (OVX CaCO_3_); (iv) OVX with nacre (OVX Nacre). Rats were anesthetized by 2.5% isoflurane and bilateral ovariectomy (OVX) was then performed via two ventro‐lateral incisions. The sham operation consisted of pulling the ovaries out of the abdominal cavity after skin incision, placing them back in the abdominal cavity, and suturing. No postoperative complications were observed.

Both Sham and OVX groups received standard diet purchased by SAFE diets (containing 8.5 mg calcium/g food) and the last two groups received the standard diet mixed with 0.25% supplemental CaCO_3_ or nacre powder. The diet was initiated on the day of surgery and was maintained for a period of 28 days after OVX. *Pinctada maxima*'s nacre powder provided by Stansea, which is composed of 97% CaCO_3_ (ie, 38% calcium) was compared with aragonite CaCO_3_ (ie, 40% calcium) synthesized according to the protocol of Carteret and colleagues.^(^ ^22^ ^)^ Calcium content was the same for both the CaCO_3_ and nacre diets and 12% higher than standard diet. The composition of the diets is shown in Supplemental Table S1.

At the end of the study, rats were injected intraperitoneally with tetracycline (25 mg/kg body weight) at days 12 and 3 before killing to allow the bone histodynamic evaluation. Animals were weighed on arrival, before OVX, and every 2 weeks during the study. All animals were killed at the age of 20 weeks using isoflurane (4%) and cardiac puncture. The uterus was dissected and immediately weighed to check OVX efficacy.

### 2.2 Bone microarchitectural analysis

**Fig. 1:**
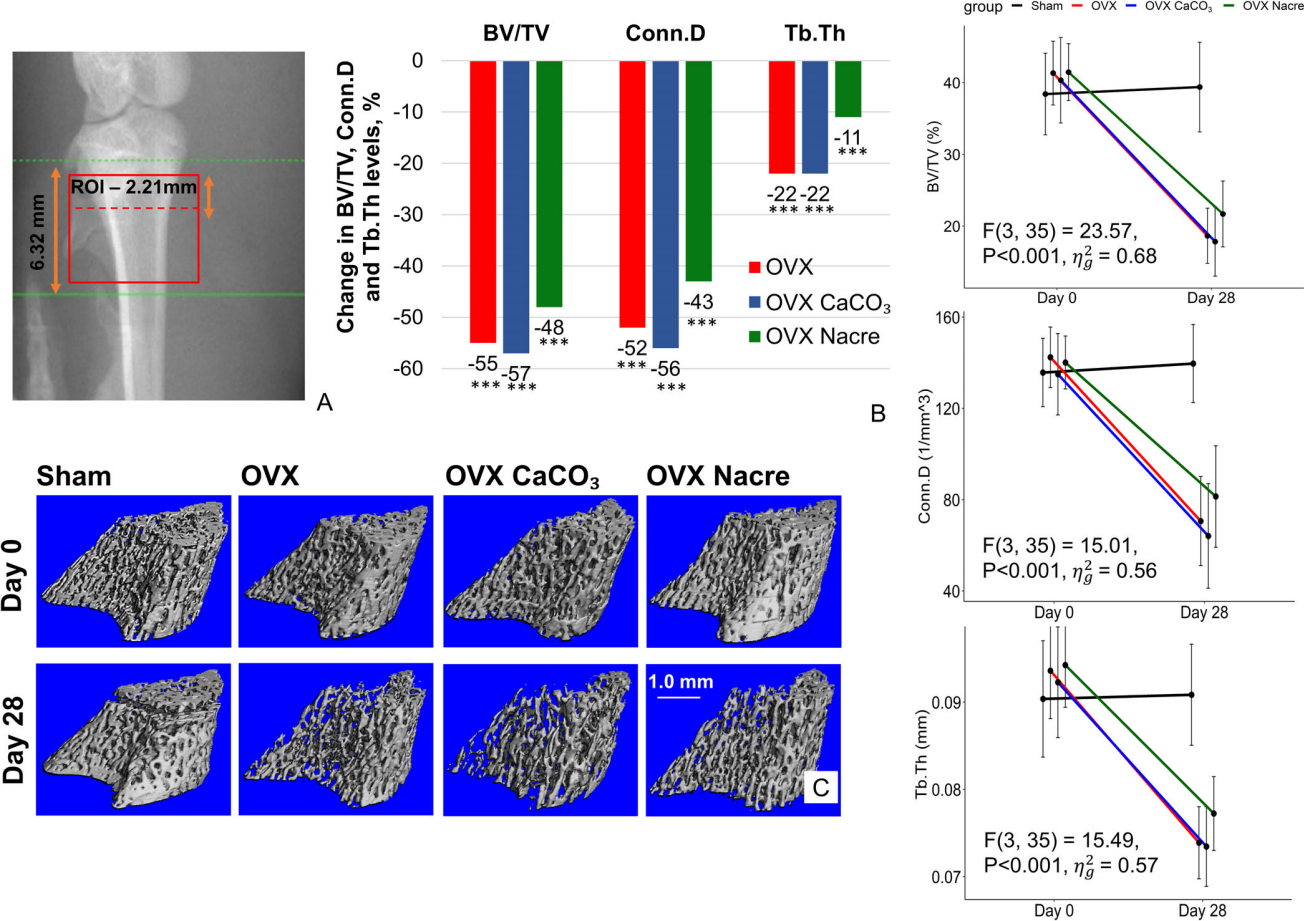
The changes of morphological trabecular bone from baseline (start point D0) to follow‐up (end point D28) in the proximal tibia in in vivo longitudinal study. (*A*) μCT scout‐view image, in which the box depicts a scanned region of 147 consecutive slices (15 μm/slice) analyzed for trabecular bone volume, as detailed in methods. (*B*) Percent changes in BV/TV, Conn.D, and Tb.Th in OVX groups compared with Sham. (*C*) Representative 3‐dimensional (3D) μCT images of the proximal tibial metaphysis of an animal (median from each group) at day 0 and day 28. Data represent the change of baseline‐adjusted mean groups relative to Sham in % (*n* = 10 per group). ^**^ *p* ≤ 0.01, ^***^ *p* ≤ 0.001, *versus Sham (ANCOVA generalized linear model using values at baseline as covariable described in the text). The raw data (right column) show the mean and 95% CIs used for ANCOVA analysis, which provide F (df_between,_ df_within)_ = test statistic, *p* values of the group effect, while adjusting for baseline and effect size generalized eta squared (ƞ_g_^2^).

We performed in vivo μCT longitudinal analysis of the proximal tibia including trabecular and cortical bone on the same acquisition by MicroCT40 (Scanco, Bruttisellen, Switzerland). The scanning system was calibrated at 70 kVp, 110 μA, and 1000 projections by 180 degrees with an integration time of 250 ms at high resolution (isotropic voxel size at 15 μm^3^ field of view (FOV)/diameter 30.7 mm). Rats were anesthetized and maintained on anesthetic isoflurane 2% for the duration of each measurement (approximately 25 minutes per rat). Tibias were scanned from the proximal growth plate and processed distally for 421 slices (6.32 mm) (Fig. 1*A*). CT Analyzer V6.6 software was used for measuring bone volume.

During the dissection, right tibias, right femora, and second lumbar spines were harvested and immediately fixed for 72 hours in 10% neutral‐buffered formalin and stored in 70% ethanol solution at 4°C. These bones were scanned by the same μCT system at 10.5 μm^3^ FOV/diameter 21.5 mm. Detailed description of scanning and evaluation procedures is provided in Supplemental Materials (Section S1 and Supplemental Table S2).

### 2.3 Bone histomorphometry

Description of the methods is provided in Supplemental Materials (Section S2).

### 2.4 RNA isolation and quantitative RT‐PCR analysis

The bone marrow and the cortical fraction of left femur were separated by flushing with a syringe and then immediately frozen in liquid nitrogen. Total RNA was extracted by TRI Reagent (Sigma, St Louis, MO, USA) and then purified with a Qiagen pure tissue kit (RNeasy Plus Mini Kit, Qiagen, Valencia, CA, USA). The RNA quality was assessed by Experion automated electrophoresis station (Bio‐Rad, Hercules, CA, USA), followed by Ribogreen assay (Quant‐iT RiboGreen RNA Assay Kit, Invitrogen, Life Technologies, Eugene, OR, USA) for accurate RNA quantification. Total RNA reverse transcription was performed with iScript cDNA synthesis kit (Bio‐Rad). Quantitative real‐time (qRT) polymerase chain reaction (PCR) was conducted on CFX96 RealTime System (Bio‐Rad) with LightCycler FastStart DNA Master plus SYBRgreen I (Roche Diagnostics, Basel, Switzerland). The mRNA expression levels were normalized to the housekeeping *Hprt1* gene expression using the ∆Ct method.^(^ ^23^ ^)^ Primer sequences are shown in Supplemental Table S3.

### 2.5 Biochemical markers in blood

Rats were fasted overnight before each blood collection by intravenous puncture in the tail at surgery day (D0: start point) and day 14 (D14: intermediate point), and by cardiac puncture under anesthesia at the end of the study (D28: end point). Blood was centrifuged at 13000 ***g*** for 2 minutes at room temperature for D0 and D14 samples or at 2000 ***g*** for 10 minutes at 4°C for D28 samples and separated in serum and plasma, then stored at −80°C. Serum levels of C‐terminal collagen cross‐linking telopeptide of type I collagen (CTX), procollagen type I N‐terminal propeptide (P1NP), and osteocalcin (Osteocalcin) were quantified by ELISA commercial kits according to manufacturer protocols (Ratlaps CTX‐I EIA kits, Rat/Mouse P1NP EIA kits, and Rat‐MID Osteocalcin EIA, Immunodiagnostic Systems, Paris, France, respectively). Serum calcium and inorganic phosphate levels were measured with colorimetric assays (Kit Biolabo SAS, Maizy, France). In addition, levels of parathyroid hormone (PTH) and fibroblast growth factor 23 (FGF23) in plasma were measured by Rat Intact PTH ELISA Kit (Immunotopics, San Clemente, CA, USA) and Mouse/Rat FGF23 Intact ELISA Kit (Immunotopics), respectively.

### 2.6 Statistical analyses

Graphical data are presented as box plots, with the central box spanning 25th to 75th percentiles and the central line representing the median. Whiskers represent the 10th and 90th percentiles and dots represent the sampling distribution. Graphs depicting body weight changes and serum/plasma biomarker levels are presented as line plots, with vertical bars depicting the mean and standard error (SE). Data were checked for normality and distribution using Quantile‐Quantile plots and Shapiro–Wilk test. Numerical tabular data are shown as the median and interquartile range (IQR) or mean and standard deviation (SD). Power analysis and calculation of sample size were performed before the initiation of the study by G*Power to define the alpha level of 0.05 and the beta level of 0.15, thereby the sample size was *n* = 10/group.

For longitudinal data analysis, (i) comparisons between groups in μCT in vivo study at follow‐up were based on analysis of covariance (ANCOVA) general linear models using values at baseline as covariable, as a standard method.^(^ ^24^ ^)^ Tukey's honest significant difference (HSD) post hoc test was then performed to report 95% confidence intervals (CI); (ii) biochemical marker levels in serum/plasma were analyzed by two‐way repeated measures ANOVA to determine if there were group effects (between‐subjects factor), time effects (within‐subjects factor), and interactions between them. After ANOVA, Tukey's HSD post hoc test was used for multiple comparisons between groups and between the value at time point versus that at previous time point in each group.^(^ ^25^ ^)^

For cross‐sectional data analysis, parameters of the μCT ex vivo bone microarchitecture, histomorphometry, and relative gene expressions were compared between groups using ANOVA, followed by Tukey's HSD post hoc test. If the normal condition was not satisfied, Kruskal–Wallis test was used instead, and Mann–Whitney–Wilcoxon (MWW) unpaired test corrected by the false discovery rate adjustment method of Benjamini and Hochberg^(^ ^26^ ^)^ was then performed for pairwise comparisons.

A nominal *p* value of 0.05 was considered statistical significance. Data analysis was conducted with R Statistical Environment and data visualization using the ggplot2 package.

The μCT and histomorphometric ex vivo variables in the tibia were processed with principal component analysis (PCA), an unsupervised method of machine learning,^(^ ^27^ ^)^ by function prcomp in base R environment and the autoscale function was applied to the data set. Graphical visualization was shown using package factoextra.

## 3 Results

### 3.1 Validation of the ovariectomy‐induced bone loss model in rat

All rats showed a gradual increase of body weight over the first 8 weeks before OVX, according to physiological growth (Supplemental Fig. S1 *A*). Beginning from 14 days post‐OVX, a weight difference of approximately 12% was observed between OVX and Sham groups (254.85 ± 8.75 versus 228.6 ± 9.82 g, *p* < 0.001). The final body weights of OVX and Sham groups at day 28 were 271.27 ± 2.25 g and 237.25 ± 9.6 g (*p* < 0.001), respectively. The uterus weight of OVX rats was significantly lower than that of Sham (*p* < 0.001) due to estrogen deficiency–induced uterine atrophy. No significant difference in uterine weight between the different OVX groups was observed (data not shown).

**Fig. 2:**
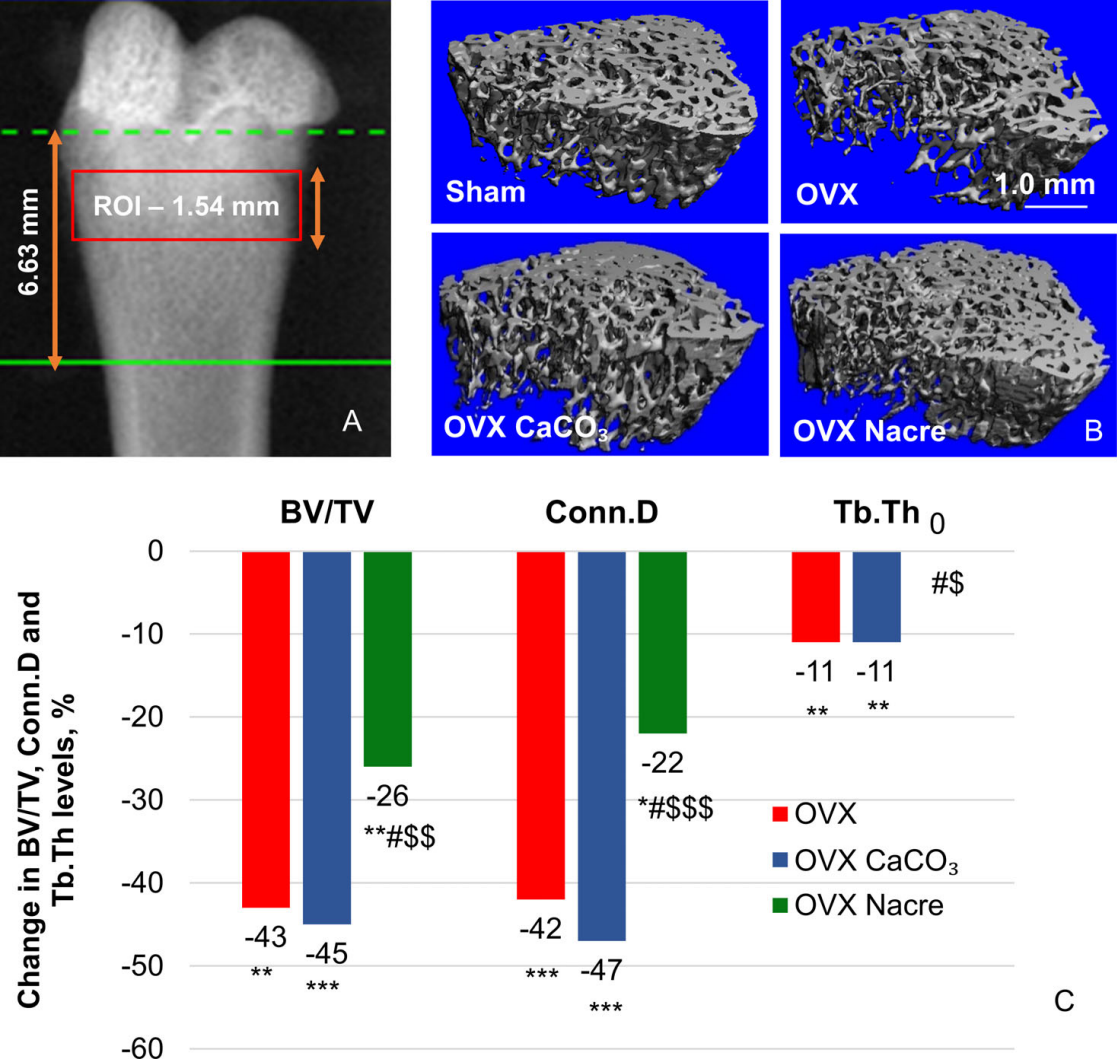
The morphological trabecular parameters in the distal metaphyseal femur in ex vivo cross‐sectional micro‐computed tomography (μCT) study. (*A*) μCT scout image, in which the box depicts a scanned region of 147 consecutive slices (10.5 μm/slice) analyzed for trabecular bone volume, as detailed in Materials and Methods. (*B*) Representative 3‐dimensional images of the trabecular bone microarchitecture in the right distal femur. (*C*) Percent changes in BV/TV, Conn.D, and Tb.Th in OVX groups compared with Sham group. Data represent the change of median OVX groups relative to Sham in % (*n* = 10 per group). *p ≤ 0.05, **p ≤ 0.01, ***p ≤ 0.001; *versus Sham group, ^#^versus OVX group, ^$^versus OVX CaCO₃ group (Kruskal–Wallis test followed by Mann–Whitney–Wilcoxon unpaired test with Benjamini–Hochberg adjustment). Sample distributions were shown in Supplemental Fig. S3.

The trabecular microarchitectural parameters were significantly deteriorated in OVX compared with Sham, regardless of the bone sites (Figs. 1 and 2 and Table 2). In the tibia, after accounting for baseline, trabecular bone volume/tissue volume (BV/TV) was 55% lower (18.5 versus 41.3; 95% CI, −29.1 to −13.1; *p* < 0.001), trabecular thickness (Tb.Th) was 22% lower (0.07 versus 0.09; 95% CI, −0.02 to −0.01; *p* < 0.001), and trabecular spacing (Tb.Sp) was +67% higher (0.25 versus 0.15; 95% CI 0.03 to 0.16; *p* = 0.003) in OVX group (Fig. 1 *B*, *C*; Table 1). As expected, these quantitative parameters were similarly affected in the cross‐sectional analyses (Supplemental Fig. S2).

**Table 1:** Trabecular Bone Morphology at Baseline and Follow‐Up in the Proximal Tibias in the Sham and OVX Rats (In Vivo Longitudinal Study)

Characteristic (unit)	Estimate	Group			
		Sham	OVX	OVX CaCO₃	OVX Nacre
BV/TV (%)	Baseline	38.42 (7.95)	41.34 (6.21)	40.34 (8.33)	41.47 (5.56)
	Follow‐up	39.39 (8.73)	18.64 (5.37)	17.84 (6.73)	21.69 (6.41)
	Adjusted follow‐up	41.3	18.5^***^	17.8^***^	21.4^***^
Conn.D (1/mm^3^)	Baseline	135.75 (20.96)	142.42 (18.54)	134.99 (24.92)	140.13 (16.22)
	Follow‐up	139.68 (23.88)	70.64 (27.25)	64.14 (32.0)	81.42 (31.06)
	Adjusted follow‐up	142.2	68.0^***^	63.2^***^	81.2^***^
Tb.N (1/mm)	Baseline	5.17 (0.53)	5.37 (0.44)	5.28 (0.62)	5.37 (0.33)
	Follow‐up	5.24 (0.60)	3.87 (0.60)	3.71 (0.79)	4.20 (0.68)
	Adjusted follow‐up	5.37	3.82^***^	3.71^***^	4.17^**^
Tb.Th (mm)	Baseline	0.09 (0.01)	0.09 (0.01)	0.09 (0.01)	0.09 (0.01)
	Follow‐up	0.09 (0.01)	0.07 (0.01)	0.07 (0.01)	0.08 (0.01)
	Adjusted follow‐up	0.09	0.07^***^	0.07^***^	0.08^***^
Tb.Sp (mm)	Baseline	0.16 (0.03)	0.15 (0.02)	0.16 (0.03)	0.15 (0.01)
	Follow‐up	0.16 (0.03)	0.25 (0.05)	0.27 (0.09)	0.22 (0.04)
	Adjusted follow‐up	0.15	0.25^**^	0.27^***^	0.23*
SMI^a^	Baseline	0.68 (0.86)	0.52 (0.60)	0.50 (0.74)	0.52 (0.62)
	Follow‐up	0.55 (0.90)	2.38 (0.32)	2.40 (0.38)	2.24 (0.43)
	Adjusted follow‐up	0.45	2.38^***^	2.40^***^	2.24***

OVX = ovariectomized; CaCO_3_ = calcium carbonate; BV/TV = trabecular bone volume/tissue volume; Conn.D = connectivity density; Tb.N = trabecular number; Tb.Th = trabecular thickness; Tb.Sp = trabecular spacing; SMI = structure model index.

Microarchitectural parameters were adjusted for individual values at baseline (start point) by using the analysis of covariance generalized linear model described in the text.

Values are mean (standard deviation), *n* = 10 per group.

^a^For the structure model index: 0 parallel plates, 3 cylindrical rods, and 4 spheres.

*p ≤ 0.05; ^**^ *p* ≤ 0.01; ^***^ *p* ≤ 0.001; *versus Sham.

In the femur (Fig. 2 and Supplemental Fig. S3), trabecular BV/TV was 43% lower (*p* = 0.002), trabecular number (Tb.N) was 26% lower (*p* < 0.001), Tb.Th was 11% lower (*p* = 0.01), and Tb.Sp was +53% higher (*p* < 0.001) in OVX group compared with Sham. In the second lumbar spine (Table 2), BV/TV and Tb.N were significantly lower in OVX group than in Sham (−12%, *p* = 0.05; and −6%, *p* = 0.01, respectively), with no change in Tb.Th. (*p* > 0.10).

**Table 2:** μCT‐Derived Trabecular Bone Microarchitectural Parameters in the Second Lumbar Spine (LS_2_) in Ex Vivo Cross‐Sectional Study

Characteristic (unit)	Group				*p* Value^a^
	Sham	OVX	OVX CaCO₃	OVX Nacre	
BV/TV	0.41 (0.37–0.42)	0.36* (0.35–0.39)	0.35** (0.34–0.35)	0.39^#$$^ (0.38–0.41)	**0.01**
Conn.D (1/mm^3^)	102.28 (88.38–106.24)	87.87 (85.35–96.85)	90.19 (84.24–92.17)	88.98 (79.97–94.33)	>0.10
Tb.N (1/mm)	4.79 (4.60–5.02)	4.50** (4.39–4.57)	4.25***^#^ (4.20–4.39)	4.45*^$^ (4.35–4.64)	**0.001**
Tb.Th (mm)	0.08 (0.08–0.09)	0.08 (0.08–0.08)	0.08 (0.08–0.08)	0.09 (0.08–0.09)	>0.10
Tb.Sp (mm)	0.18 (0.17–0.19)	0.20* (0.19–0.20)	0.21**^#^ (0.20–0.22)	0.20*^$^ (0.19–0.20)	**0.001**
SMI^b^	−0.74 (−0.84–0.16)	−0.29* (−0.44–0.12)	−0.21 (−0.30–0.01)	−0.52 (−0.73–0.39)	**0.03**

OVX = ovariectomized; CaCO_3_ = calcium carbonate; BV/TV = trabecular bone volume/tissue volume; Conn.D = connectivity density; Tb.N = trabecular number; Tb.Th = trabecular thickness; Tb.Sp = trabecular spacing; SMI = structure model index.

Post hoc pairwise comparisons were conducted using Mann–Whitney‐Wilcoxon (MWW) unpaired tests with Benjamini–Hochberg adjustment.

Values are median (interquartile range), n = 10 per group.

The *p* values in bold indicate statistical significance at level of 5% (*p* ≤ 0.05).

^a^Kruskal–Wallis test.

^b^For the structure model index, 0 indicates parallel plates, 3 cylindrical rods, and 4 spheres.

*p ≤ 0.05; **p ≤ 0.01; ***p ≤ 0.001; *versus Sham; ^#^versus OVX; ^$^versus OVX CaCO₃.

We also assessed the cortical bone microarchitecture in the diaphysis of both tibia and femur, but no change was roughly observed at day 28 post‐OVX (Supplemental Table S4).

**Fig. 3:**
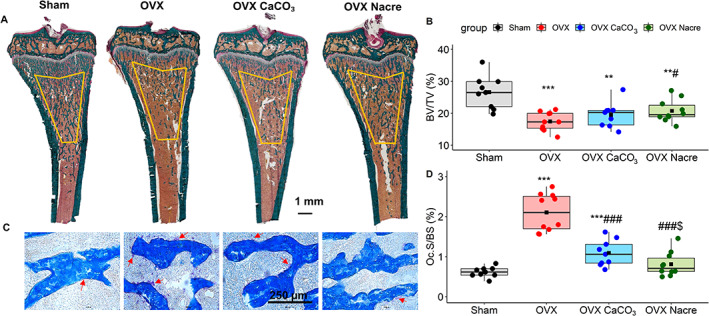
The histomorphometric characteristics in the undecalcified proximal metaphyseal tibia through 9 μm sections by day 28. (*A*) Representative histological sections at day 28 from the undecalcified proximal metaphyseal tibia stained with Goldner's trichrome. They show full longitudinal sections of median animals in each group and the region of interest (ROI) for histomorphometry measurements is outlined in yellow. Scale bar = 1 mm. **(B)** The bone volume fraction (BV/TV) derived from sections stained with Goldner's. **(C)** Representative sections stained with tartrate – resistant acid phosphate (TRAcP). The red arrows indicate red‐stained osteoclasts, scale bar = 250 μm. **(D)** The osteoclast surface per bone surface parameter (Oc.S/BS) derived from sections stained with TRAcP. Data represent as boxplots, and show all data points, with interquartile range (IQR) (height of the box), median (internal horizontal bar) *n* = 9 per Sham / OVX CaCO_3_ group; *n* = 10 per OVX / OVX Nacre group), mean (black filled square, ▪). *p ≤ 0.05, **p ≤ 0.01, ***p ≤ 0.001; * *vs*. Sham, ^#^ *vs*. OVX, ^$^ *vs*. OVX CaCO_3_ (Kruskal‐Wallis test followed by Mann‐Whitney‐Wilcoxon unpaired test with Benjami‐Hochberg adjustment).

Representative images and parameters for histomorphometry evaluation in longitudinal sections of non‐decalcified tibias are shown in Fig. 3 and Table 4. As expected, OVX induced a significant increase in bone remodeling with a 3.4‐fold increase of osteoclast surface and 3‐fold increase in bone formation rate (*p* < 0.001) compared with Sham.

**Fig. 4:**
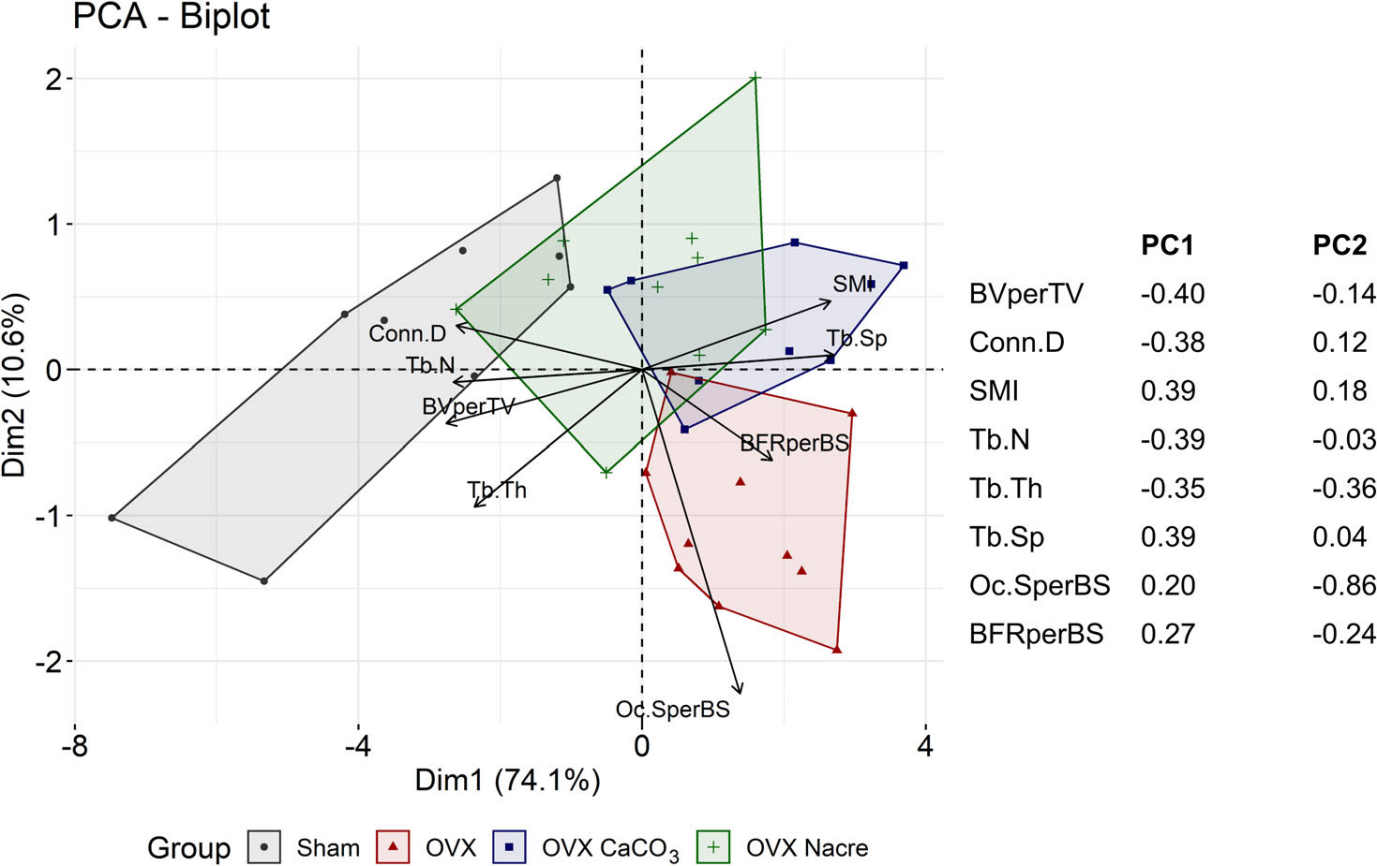
Principal component analysis (PCA) in the tibia for trabecular variables measured by μCT and histomorphometry. Biplot includes score plots that represent the distribution of individual rats in the space defined by principal component 1 (PC1) and PC2, and loading plots that represent the contribution of each variable, corresponding to each loading vector in the space defined by PC1 and PC2. The weights of loading vectors in each PC are shown in the right panel. *n* = 9 per Sham/OVX CaCO_3_ group; *n* = 10 per OVX/OVX Nacre group.

The global visualization among the μCT and histomorphometry variables in tibia and the rats was explored with PCA (Fig. 4). Score plot shows two separated clusters that were formed by Sham group (in black) and OVX group (in red) along the first component. This component explained up to 74.1% variance of all of variables. The first loading vector places most of its weight on Tb.Sp, structure model index (SMI), bone formation rate/bone surface (BFR/BS) and osteoclast surface/bone surface (Oc.S/BS) and much less weight on the other four features. Hence, this first component roughly corresponds to the level of trabecular quantification and bone remodeling activity of the rats.

**Fig. 5:**
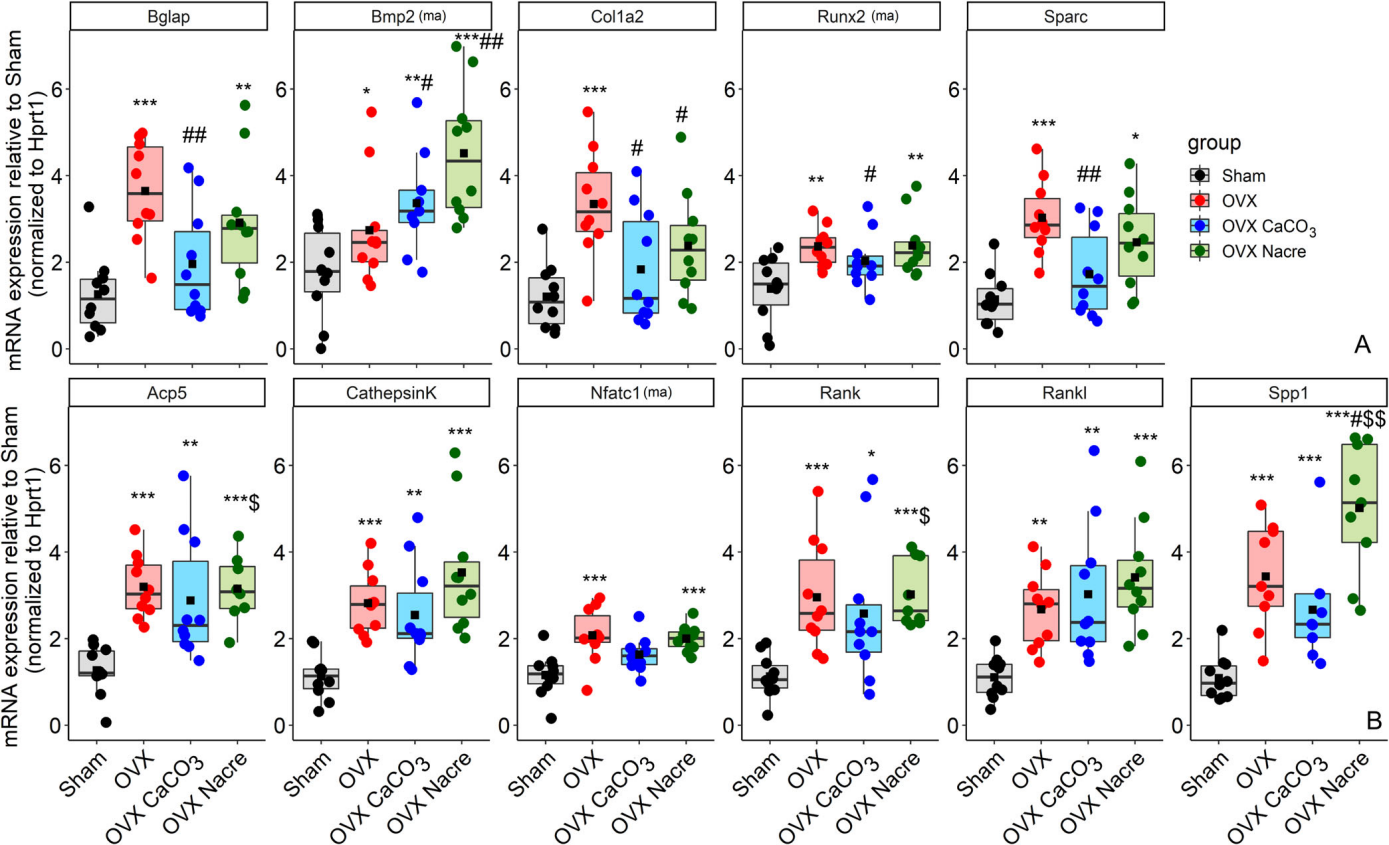
mRNA expression levels of genes involved in bone formation (*A*) and bone resorption (*B*) using extracts from flushed marrow (ma) or bone fraction at day 28. Transcript levels were normalized to housekeeping gene *Hprt1* following the 2^(−∆∆C(t))^ method.^(^ ^23^ ^)^ The specific primers (based on the rat sequences) are displayed in Supplemental Table S1. Data represent as box plots and show all data points, with interquartile range (IQR) (height of the box), median (internal horizontal bar) (*n* = 10 per group), mean (black filled square, ■). *p ≤ 0.05, **p ≤ 0.01, ***p ≤ 0.001; *versus Sham, ^#^versus OVX, ^$^versus OVX CaCO₃ (ANOVA test followed by Tukey's HSD post hoc test or Kruskal–Wallis test followed by Mann–Whitney–Wilcoxon unpaired test with Benjami–Hochberg adjustment). *Hprt1 =* hypoxanthine phosphoribosyltransferase 1.

In line with the μCT and histomorphometry results, estrogen deficiency also induced an increase of mRNA expression of gene markers involved in both osteoblastogenesis and osteoclastogenesis. As shown in Fig. 5, OVX rats exhibited the higher amounts of *Bgalp* (3‐fold, *p* < 0.001) and *Runx2* mRNA (+1.70‐fold, 2.37 versus 1.39; 95% CI, 0.20 to 1.76; *p* = 0.009) and both approximately 2‐fold higher *NFATc1* (2.08 versus 1.16; 95% CI, 0.35 to 1.49; *p* < 0.001) and *Rank* (*p* < 0.001) compared with Sham group.

**Fig. 6:**
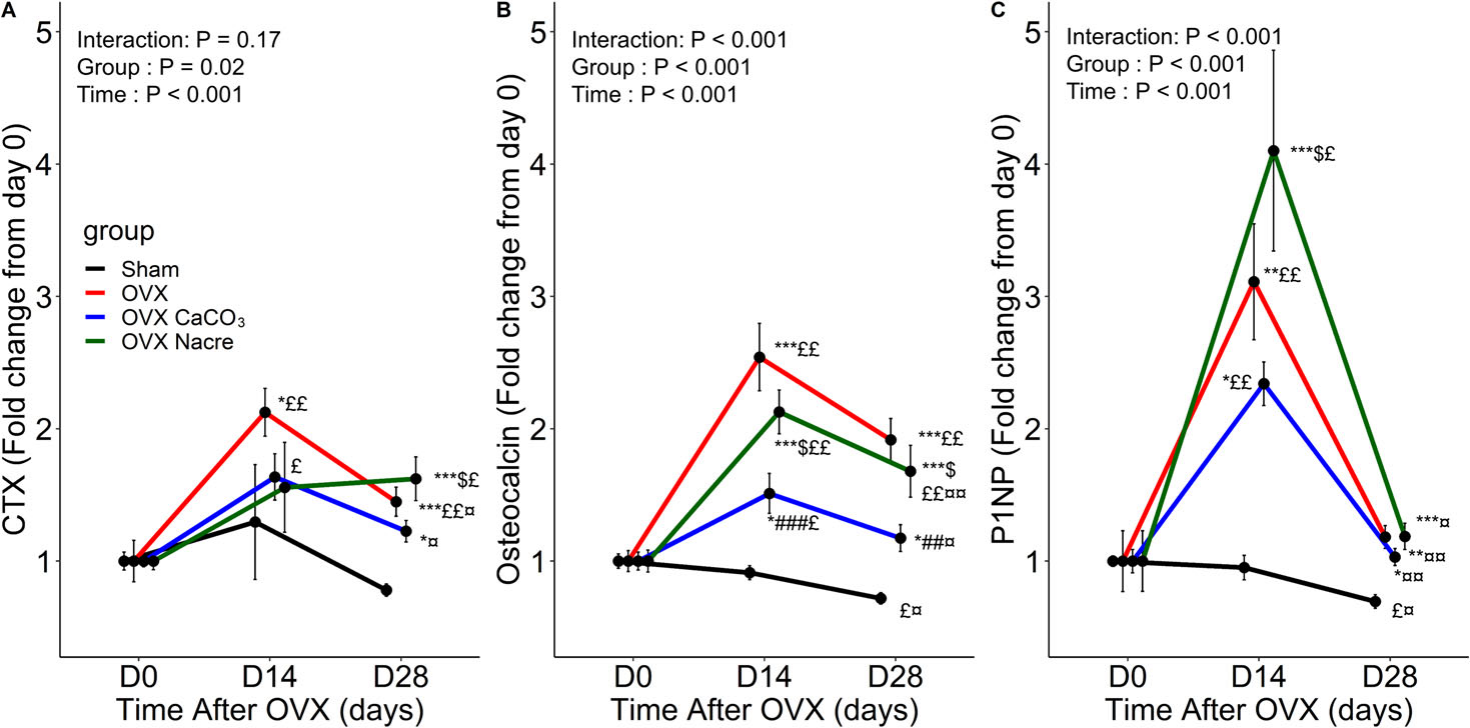
Changes of serum bone turnover markers from baseline (day 0) to follow‐up in the Sham and OVX rats. The data were represented as the mean ± standard error (vertical bar) (*n* = 6 per group). The *p* values indicated the effect of group (between‐subject factor) and/or time (within‐subject factor) and the group × time interaction using two‐way repeated measures ANOVA, followed by Tukey's HSD post hoc test to adjust for multiple comparisons. ^#^ *p* ≤ 0.05, ^##^ *p* ≤ 0.01, ^###^ *p* ≤ 0.001, *versus Sham, ^#^versus OVX, ^$^versus OVX CaCO_3_; ^£^versus baseline at day 0, ^¤^versus follow‐up at day 14.

In agreement with the above results, the serum levels of bone turnover biomarkers were significantly elevated in OVX rats with respect to time (Fig. 6). There were sustained increases in osteocalcin and CTX levels over time, whereas PINP level showed a transient increase at day 14 and resumed baseline values thereafter. Sham rats gradually declined the secretion of these markers over time (Fig. 6).

### 3.2 Effect of nacre against weight gain and bone loss in comparison to standard and CaCO_3_ diet in OVX rats

#### 3.2.1 Body weight

Because of the significant differences in body weight between groups observed at the beginning of study (*p* = 0.04) (Supplemental Fig. S1 *A*), we transformed the raw data set into symmetric percent change from baseline values before conducting pairwise comparisons between OVX groups (Supplemental Fig. S1 *B*). From baseline, nacre diet significantly limited body weight gain by 44% (6.5 versus 11.6; 95% CI, −8.1 to −2.1; *p* < 0.001) and 38% (6.1 versus 10.5; 95% CI, −7.0 to −0.92; *p* = 0.006) compared with OVX and OVX CaCO_3_ group, respectively, at day 14. Both CaCO_3_ and nacre diet reduced by 22% of weight at the end of the study (*p* = 0.02).

#### 3.2.2 Bone microarchitecture analysis

In the in vivo longitudinal survey of tibia metaphysis, bone volume BV/TV was lower 16% for the OVX group (−2.9; 95% CI, −11.0 to 4.93; *p* > 0.10) and 20% (−3.6; 95% CI, −11.7 to 4.2; *p* > 0.10) for OVX CaCO_3_ group compared with OVX Nacre group in a large effect size (${ƞ}_{g}^{2}$ > 0.40), with mean percent change from baseline in BV/TV of −55% (OVX), −56% (OVX CaCO_3_), and −48% (OVX Nacre). Supplementing with nacre also led to higher connectivity density (Conn.D), Tb.N, and Tb.Th and to lower Tb.Sp compared with those of both standard and CaCO_3_ diets, after accounting for baseline values (Table 1 and Fig. 1). Also, ex vivo cross‐sectional evaluation performed at a higher resolution showed similar significant positive effect of nacre on bone mass and microarchitecture (Supplemental Fig. S2), whereas CaCO_3_ diet did not show any protective impact on quantitative trabecular parameters in tibia.

In the femur distal metaphysis (Fig. 2 and Supplemental Fig. S3), trabecular bone mass and microarchitecture parameters of OVX CaCO_3_ were not different from those of the OVX group. In contrast, nacre diet prevented trabecular BV/TV loss by about 40% compared with standard (*p* = 0.04) and CaCO_3_ (*p* = 0.004) diets in OVX rats, as well as other microarchitectural parameters.

The OVX‐induced deterioration of microarchitecture in the second lumbar spine was also moderately prevented by nacre diet (Table 2). In nacre‐fed OVX rats, the bone volume was significantly higher compared with standard (+8%, *p* = 0.04) and CaCO_3_ (+11%, *p* = 0.01) diets, and trabecular number and separation were equal to standard but better than CaCO_3_ diet (*p* = 0.03 and 0.02, respectively). The protective effects of nacre in trabecular bone at various bone sites are summarized in Table 3 by comparing with standard and CaCO_3_ diets in OVX rats.

**Table 3:** Summary of Representative Parameters of Trabecular Bone Analysis in Different Skeletal Sites Using Ex Vivo μCT

Bone site	Trabecular parameters	OVX Nacre versus OVX^#^	OVX Nacre versus OVX CaCO_3_^$^
Femoral distal metaphysis	BV/TV	0.07 (**0.04** ^#^)	0.08 (**0.004** ^$$^)
	Tb.Th (mm)	0.01 (0.10)	0.01 (**0.03** ^$^)
	Tb.N (1/mm)	0.54 (**0.03** ^#^)	0.93 (**0.002** ^$$^)
	Tb.Sp (mm)	−0.03 (**0.05** ^#^)	−0.07 (**0.002** ^$$^)
Second lumbar spine (LS_2_)	BV/TV	0.03 (**0.04** ^#^)	0.04 (**0.01** ^$$^)
	Tb.Th (mm)	0.01 (>0.10)	0.01 (>0.10)
	Tb.N (1/mm)	−0.05 (>0.10)	0.20 (**0.03** ^$^)
	Tb.Sp (mm)	0.00 (>0.10)	−0.01 (**0.02** ^$^)
Tibial proximal metaphysis	BV/TV	0.01 (0.09)	0.06 (0.07)
	Tb.Th (mm)	0.01 (>0.10)	0.01 (**0.05** ^$^)
	Tb.N (1/mm)	0.18 (>0.10)	0.52 (**0.01** ^$$^)
	Tb.Sp (mm)	0.00 (>0.10)	−0.02 (**0.05** ^$^)

OVX = ovariectomized; CaCO_3_ = calcium carbonate; BV/TV = trabecular bone volume/tissue volume; Tb.Th = trabecular thickness; Tb.N = trabecular number; Tb.Sp = trabecular spacing; SMI = structure model index.

Data were represented as difference of median and *p* values in parentheses () using Mann–Whitney–Wilcoxon unpaired tests with Benjamini–Hochberg adjustment.

The *p* values in bold indicate statistical significance at level of 5% (*p* ≤ 0.05).

^#^ *p* ≤ 0.05; ^##^ *p* ≤ 0.01; ^#^versus OVX; ^$^versus CaCO_3_.

#### 3.2.3 Bone histomorphometry evaluation

In pairwise comparisons between the three OVX groups (Fig. 3 and Table 4), the osteoclast surface was significantly decreased in both OVX CaCO_3_ (−2‐fold, 1.06 versus 2.11, *p* < 0.001) and OVX Nacre (−3‐fold, 0.71 versus 2.11, *p* < 0.001) compared with that of OVX group. Interestingly, this decrease was 1.50‐fold greater in nacre than in CaCO_3_ diet (*p* = 0.03). Bone formation parameters in OVX CaCO_3_ and Nacre groups remained as increased as in the OVX group compared with Sham.

**Table 4:** Histomorphometry of Trabecular Bone in the Proximal Tibias of Sham and OVX Rats

Type of index (unit)	Group (no. of rats)				*p V*alue^a^
	Sham (9)	OVX (10)	OVX CaCO₃ (9)	OVX Nacre (10)	
**Static indices**					
N.Oc/B.Ar (c/mm^2^)	38.99 (36.21–43.10)	122.11*** (103.22–133.25)	68.60**^###^ (44.76–75.39)	46.11^###^ (42.02–62.08)	**<0.001**
N.Oc/B.Pm (c/mm)	0.80 (0.76–0.85)	2.41*** (2.16–2.71)	1.44**^##^ (1.03–1.64)	0.98^###^ (0.78–1.34)	**<0.001**
Oc.S/BS (%)	0.62 (0.54–0.70)	2.11*** (1.70–2.51)	1.06***^###^ (0.84–1.31)	0.71^###$^ (0.63–0.97)	**<0.001**
Oc.Le (μm)	15.82 (15.33–17.52)	19.38 (16.34–18.97)	16.94 (15.34–18.01)	16.30 (15.14–17.41)	>0.10
**Dynamic indices**					
sLS/BS (%)	1.97 (1.77–2.40)	6.29** (3.51–8.19)	6.17*** (3.87–6.93)	5.42*** (4.71–5.89)	**<0.001**
dLS/BS (%)	0.70 (0.53–1.23)	4.22** (3.38–5.86)	4.23* (0.67–6.47)	3.62 (1.32–5.09)	**0.02**
MAR (μm/d)	1.34 (1.29–1.49)	1.44 (1.01–1.62)	1.74^#^ (1.63–1.91)	1.45*^$^ (1.36–1.52)	**0.04**
MS/BS (%)	1.74 (1.32–2.60)	7.73*** (5.39–9.37)	6.07*** (4.26–9.56)	6.17*** (2.32–7.73)	**<0.001**
BFR/BS (μm^3^/μm^2^/d)	3.12 (2.84–3.76)	9.20*** (6.30–10.91)	7.81*** (6.94–11.37)	7.68*** (4.97–9.24)	**<0.001**

OVX = ovariectomized; CaCO_3_ = calcium carbonate; N.Oc/B.Ar = Osteoclast Number per Bone Area; N.Oc/B.Pm = Osteoclast Number per Bone perimeter; Oc.S/BS = Osteoclast Surface per Bone Surface; Oc.Le = Osteoclast Length; sLS/BS = single‐labeled surface per bone surface; dLS/BS = double‐labeled surface per bone surface; MAR = mineral apposition rate; MS/BS = Mineralizing Surface per Bone Surface; BFR/BS = bone formation rate/bone surface.

Post hoc pairwise comparisons were conducted using Mann–Whitney–Wilcoxon unpaired tests with the Benjamini–Hochberg adjustment.

Values are median (interquartile range).

The *p* values in bold indicate statistical significance at level of 5% (*p* ≤ 0.05).

^a^Kruskal–Wallis test.

*p ≤ 0.05; **p ≤ 0.01; ***p ≤ 0.001; *versus Sham; ^#^versus OVX; ^$^versus OVX CaCO₃.

As illustrated in Fig. 4, PCA analysis showed that the OVX rats under CaCO_3_ diet exhibited similar features to those under standard diet, whereas most of the OVX rats under nacre diet close to zero score of the first component. On the other hand, OVX Nacre rats tended to exhibit Sham's trabecular characteristics and were clearly apart from OVX rats.

#### 3.2.4 Analysis of gene expression levels

To investigate the potential molecular mechanisms underlying the effect of experimental diets (CaCO_3_ and nacre) in OVX rats on osteoblastogenesis, we examined various signaling factors involved in osteoblast differentiation or reflecting its activity. Despite a global decrease in expression of the bone formation–related genes such as *Col1a1*, *Bglap*, and *Runx2* in OVX CaCO_3_ and nacre groups compared with OVX, the mRNA amounts of these genes were more expressed in nacre than in CaCO_3_ (Fig. 5*A*). By contrast, *Bmp2* mRNA levels were upregulated by 1.30‐fold in CaCO_3_ (*p* = 0.05) and by 1.70‐fold in nacre diet (*p* = 0.004) compared with standard diet in OVX rats.

Unexpectedly, while CaCO_3_ reduced the mRNA expression of almost all osteoclastogenesis‐related genes compared with standard diet in OVX rats, nacre supplementation significantly upregulated that of *Spp1* by 2.20‐fold (*p* = 0.01) and *Acp5* by 1.45‐fold (*p* = 0.04) compared with the CaCO_3_ diet, even more than standard diet in some genes, *Cathepsin K* (+1.25‐fold; 3.53 versus 2.82; 95% CI, −0.53 to 1.95; *p* > 0.10) and *Spp1* (+1.45‐fold, *p* = 0.05) (Fig. 5*B*).

#### 3.2.5 Bone turnover biomarker measurements

With regard to changes in blood biomarkers over time, the between‐groups test indicated that the variable group was significant; consequently, in the graph we see that the lines for four groups are rather far apart. The within‐subjects test indicated that there was a significant time effect; in the other words, each group did change over time. Moreover, the interaction of time and group was significant, which means that the groups were changing over time but were changing in different ways (Fig. 6).

At day 14, CaCO_3_ and nacre limited the OVX‐induced increase in both serum osteocalcin and CTX levels. Interestingly, P1NP levels increased more under nacre (+4‐fold, *p* = 0.02) than under CaCO_3_ diet (+2.30‐fold, *p* = 0.004), compared with baseline. Osteocalcin levels from baseline were similarly increased in nacre (+2.10‐fold, *p* = 0.001) and CaCO_3_ (+1.50‐fold; *p* = 0.03). At day 28, CaCO_3_ diet diminished the fluctuation of three markers related to OVX (*p* < 0.05 or less, compared with day 14) but still more elevated than those of Sham (*p* < 0.05). In contrast, nacre showed more abundance of osteocalcin and CTX than CaCO_3_ (*p* = 0.02) and illustrated by approximately +1.50‐fold or more versus baseline values.

We next examined whether nacre might potentially affect the calcium – phosphate homeostasis regulated by the estrogen – PTH – FGF23 axis. As shown in Fig. S5, circulating PTH change from baseline was not different at day 14 in both OVX and OVX Nacre groups and then dramatically went up to 3.6‐fold higher in OVX group at day 28 (*p* = 0.05), whereas that of nacre was not detected.

Nacre supplements also had a small but significant effect on serum phosphate, with a transient about 1.50‐fold increase from baseline at day 14 and 28 (*p* = 0.02), which were not associated with significant changes in serum FGF23 levels.

## 4 Discussion

The products of natural origin could play a role in novel preventative strategies against osteoporosis considered as a major health problem for postmenopausal women in the community.^(^ ^6^, ^9^, ^10^ ^)^ The properties of nacre and its matrix factors on bone structure and quality were examined in in vitro and in vivo studies.^(^ ^8^, ^14^, ^18^ ^)^ Here, we provide the additional evidence for nacre‐based beneficial effects in OVX rats, as an appropriate preclinical model for postmenopausal bone loss.

### 4.1 Bone morphology and bone turnover changes related to ovariectomy in brief

#### 4.1.1 Bone microarchitecture

It is recognized that the bone growth has slowed considerably but not stopped in mature rat model.^(^ ^19^ ^)^ However, this accelerated bone growth is only transient, and the final observed trabecular parameters of OVX rats were lower than those of Sham rats that true bone loss occurred (Fig. 1, right column). In the other words, the negative influence of estrogen deficiency on bone balance is so dominant in the mature rat model that ovariectomy causes a marked depletion of trabecular bone in the appendicular skeleton despite of any residual growth.

SMI parameter in μCT evaluation has been widely used as an indicator of the structure of trabeculae. SMI will be 0 for parallel plates and 3 for cylindrical rods.^(^ ^28^ ^)^ However, our μCT findings presented both negative and positive values, particularly for Sham group in the tibial (Supplemental Fig. S2), femoral metaphysis (Supplemental Fig. S3), and all groups in the lumbar spine (Table 2). The previous work mentioned that the bone surface processes both convex and concave regions, which have been measured by positive and negative SMI, respectively. The final SMI value is the sum of all components.^(^ ^29^ ^)^ Also, this study demonstrated the negative strong correlation between BV/TV and SMI in the rat model. This suggestion should aid in explaining why the bone loss in OVX groups linked to the plate‐to‐rod transition of trabeculae, which resulted in the positive SMI in some bone sites, responded to OVX after 28 days.

As expected, we found that ovariectomy in rats caused an initial rapid phase of trabecular bone loss and deterioration of bone microstructure in the metaphysis of both proximal tibia (Table 1, Fig. 1, and Supplemental Fig. S2) and distal femur (Fig. 2 and Supplemental Fig. S3), whereas cortical bone was not yet affected after 28 days post‐OVX, as reported in previous studies.^(^ ^19^, ^30^, ^31^ ^)^

#### 4.1.2 Bone histomorphology

We performed the histomorphometry analysis in the proximal tibia by Goldner's trichrome and TRAcP staining (Table 4). We found that the ovariectomy resulted in an increase of osteoclast surface and number, also an augmented bone formation rate, as demonstrated in previous results.^(^ ^32^, ^33^, ^34^ ^)^ These findings confirmed that the increment of bone turnover occurred in the early period after estrogen depletion.

#### 4.1.3 Bone turnover markers

Concerning the gene markers of bone turnover process, Choi and colleagues reported that the relative mRNA expression levels of osteoblast‐specific genes were decreased and those of osteoclast were increased in estrogen‐deficient rats after 12 weeks post‐OVX.^(^ ^32^ ^)^ By contrast, another study indicated the elevation of all analyzed gene expression after 10 weeks.^(^ ^35^ ^)^ The findings from the second study are consistent with our outcomes (Fig. 5), whereby both bone formation and resorption process are considerably activated, caused by the ovarian function loss in our rats suffered 28 days post‐OVX.

Concerning the serum markers of bone homeostasis, osteocalcin is the bone turnover marker, P1NP is a more sensitive biomarker of bone formation in osteoporosis, and CTX is a specific and sensitive biomarker of bone resorption.^(^ ^36^ ^)^ Ovariectomy significantly increased the level of these markers, reaching a peak after 14 days. In particular, P1NP had the highest change at day 14 and decline thereafter, despite still being elevated. Otherwise, osteocalcin and CTX were continuously elevated (Fig. 6 and Supplemental Fig. S5 *A*). This is correlated to the gene expression analysis that reflects the cellular activities at day 28. Our outcomes support the argument that osteopenia is detected in OVX rat as soon as 14 days post‐OVX and became progressively more pronounced up to 100 days.^(^ ^34^ ^)^ We additionally suggest that bone formation could be maximized in the initial rapid phase at day 14, then declined at later times post‐OVX, whereas bone resorption would be sustained in the longer time, so that the increase in bone resorption exceeds the increase in bone formation.

### 4.2 Beneficial effects of nacre in stimulated bone loss and bone turnover context

#### 4.2.1 Limitation of body weight gain due to loss of ovarian function as early as 14 days

Although both CaCO_3_ and nacre diets limited body weight gain after 28 days post‐OVX, this positive impact was detected as early as at 14 days with nacre, whereas that of CaCO_3_ was not (Supplemental Fig. S1). Wronski and colleagues suggested only partial protective effect of obesity against bone loss due to ovarian hormone deficiency and the osteopenia develops regardless of body weight.^(^ ^37^ ^)^ Moreover, the relationships between calcium intake and body weight are still obscure. Indeed, low calcium intake is associated with higher body weight in women.^(^ ^38^ ^)^ By contrast, the interventional trials of calcium supplementation failed to show any effect on body weight and body fat.^(^ ^39^ ^)^ Thus, the effect against weight gain of nacre should be accounting to its organic matrix content, which is measured at 2.7 wt% in *Pinctada maxima* nacre.^(^ ^40^ ^)^ Because the mechanism of body weight gain compensated for bone loss associated with OVX in rats is still unclear, the diminished body weight of nacre should be considered as a factor or a consequence explained to its protective bone effect, which is discussed below.

#### 4.2.2 Protective effect of nacre on the altered trabecular morphology 28 days after OVX

In appendicular bones (tibia and femur) and lumbar spine, nacre diet partially preserved trabecular bone mass and microarchitecture parameters (Figs, 1 and 2 and Table 2), whereas CaCO_3_ did not limit this deterioration at these bone sites at day 28. A study of Gala and colleagues reported that 15‐week‐old ovariectomized rat supplemented with calcium at dose 15 and 6.6 g/kg food during 13 and 28 weeks produced an increase on femur bone mineral density (BMD) but any significant differences were detected in both young and old animals and those were not on lumbar spine BMD.^(^ ^41^ ^)^ Here, we used only 12% higher calcium in CaCO_3_ and nacre diets during 4 weeks. Thus, these recognized preventive impacts on bone sites suggest that nacre effect could be the result not only from calcium carbonate but also from organic compounds (eg, proteins,^(^ ^14^, ^17^ ^)^ lipid,^(^ ^42^ ^)^ water‐soluble matrix,^(^ ^15^, ^18^ ^)^ ethanol‐soluble matrix^(^ ^16^ ^)^), which are known for their bioactive properties (eg, acceleration of osteoblastogenesis and inhibition of osteoclastogenesis) and also meaningfully participate in the physiopathological condition in OVX rat model.

In addition, nacre diet significantly reduced the surface and number of osteoclasts better than CaCO_3_ diet (Table 4). Therefore, our results of bone morphology and cellular histomorphometry suggested that the better and earlier protective effect on bone structure under nacre diet was associated with a greater decrease in the number of osteoclasts compared with CaCO_3_, whereas no significant differences in bone formation rate was observed between these two experimental diets at day 28. This could be attributed to an improved intestinal calcium absorption of nacre that would result in lower severity of secondary hyperparathyroidism observed at the latest time point in the OVX group. Unless, it was shown that calcium carbonate intestinal absorption is poorer than that of another organic calcium source.^(^ ^43^ ^)^ Thus, this explanation reinforces the beneficial effect derived from the organic matrix of nacre. In this regard, nacre inhibits the negative bone remodeling balance better than CaCO_3_ 28 days post‐OVX in rats.

#### 4.2.3 Modification of elevated bone turnover in a different way from that of CaCO_3_

We found that nacre diet enhanced the mRNA expression of genes involved in both osteoblastogenesis and osteoclastogenesis. Previous in vitro studies indicated that nacreous organic matrix induced osteoblast differentiation^(^ ^16^ ^)^ and suppressed osteoclast activity.^(^ ^18^ ^)^ However, the present study was realized in OVX rat model and the gene expression levels were only analyzed on femur samples at day 28. Indeed, the findings in this part should be interpreted in the context of longitudinal study. In other words, thanks to repeated biomarker measurements at three time points, each individual acts as its own control and shows its change with respect to time.

Regarding the bone turnover markers, the responses of CaCO_3_ and nacre diets to ovarian function loss were different. CaCO_3_ attenuated the stimulated bone turnover process in both bone formation and resorption, whereas nacre regulated the imbalanced bone homeostasis, in favor of elevating bone formation and slowing bone resorption at the initial rapid stage of 14 days and tending to moderately maintain the increased bone turnover, at least up to 28 days in this study, before transferring to the steady state remodeling in ovariectomy intervention. Bone turnover markers are referred as formation and resorption markers under the assumption that these are surrogates of the cellular events. Indeed, our biomarkers—related findings could explain why the gene markers of osteoblastogenesis and osteoclastogenesis‐were expressed in a more abundant manner in nacre than in CaCO_3_ diet at day 28_._ Overall, nacre may have provided an additional stimulus for bone formation that compensates bone resorption, matching with lower bone resorption after 14 days post‐OVX, that leads to more protection against bone loss.

Previous studies have described that calcium level depends on the progression and severity of deficient estrogen situation and is regulated by several factors, including parathyroid hormone (PTH).^(^ ^44^ ^)^ One of the major outcomes in this study is that the estrogen deficiency led to a fluctuated plasma PTH level at the second time, day 14 to 28 post‐ovariectomy. Nacre supplementation is capable of maintaining the stability of PTH level (Supplemental Fig. S5). Additionally, fibroblast growth factor 23 (FGF23) is known as a bone‐derived phosphaturic hormone, synthesized by osteoblasts and osteocytes, that acts directly on the parathyroid glands through the MAPK pathway to decrease serum PTH.^(^ ^45^ ^)^ It has also been suggested that the indirect effect of estrogen on PTH may require the participation of FGF23.^(^ ^46^ ^)^ Despite no marked changes in FGF23 levels in our study on this basis, we can hypothesize, in restriction to relation between bone metabolism and PTH, that nacre could impact this function through activating osteoblasts.

Our study has several strengths. First, instead of using the gavage^(^ ^7^, ^8^ ^)^ or injection^(^ ^10^, ^11^ ^)^ method used in previous studies, we gave priority to prepare the diets supplemented with CaCO_3_ or nacre close to the 3R principles of animal welfare. Additionally, we addressed the responses to bone loss of different skeletal sites, including appendicular and axial bones, which have a significant translational relevance. Also, by combining both longitudinal and cross‐sectional study designs, we generated an overall interpretation for estrogen deficient–induced bone loss in rats and thus tried to explain how nacre could protect bone structure in live animals.

Our study also has limitations. The rat skeleton should be exposed to a pharmaceutical agent for a duration either equivalent to bone turnover rates in humans (100 to 200 days) and rats (40 days),^(^ ^47^ ^)^ or at least of six remodeling cycles to allow relevant inference on long‐term bone efficacy and safety in humans.^(^ ^48^ ^)^ Although the rat has been recommended to be used as a rodent postmenopausal model, it shows a potential drawback; that is, the lack of Haversian remodeling in the intracortical bone.^(^ ^20^ ^)^ Lastly, the lack of control for dietary intake of the animals induced the inability of calculation about the average exposure of kg body weight to experimental powders.

In this study, we found that supplementation with nacre could effectively limit bone loss induced by estrogen deficiency after ovariectomy in rat better than CaCO_3_. This occurred by modulating the negative imbalance of bone turnover that led to these changes. This effect might be driven by the limitation of secondary hyperparathyroidism. However, we cannot rule out an additional uncoupling effect of nacre on bone remodeling at day 14, which could slightly stimulate bone formation. Further preclinical studies based on longer‐term evaluation and on the non‐rodent model are still needed to confirm the efficacy and safety of nacre. It remains to identify the precise mechanisms whereby the nacreous organic matrix acts on bone.

## Disclosures

MR provides scientific consultation for Megabiopharma. TT reports receiving lecture fees from Amgen, Arrow, Biogen, BMS, Chugai, Galapagos, Grunenthal, Jansen, LCA, Lilly, MSD, Nordic, Novartis, Pfizer, Sanofi, Thuasne, Theramex, and UCB and research grants or investigator's fees from Bone Therapeutics, UC. All other authors state that they have no conflicts of interest.

## Author contributions

**Dung Kim Nguyen:** Data curation; formal analysis; methodology; visualization; writing – original draft; writing – review and editing. **Norbert Laroche:** Data curation; formal analysis; investigation; methodology. **Arnaud Vanden‐Bossche:** Conceptualization; methodology. **Marie‐Thérèse Linossier:** Data curation; formal analysis; investigation; methodology. **Mireille Thomas:** Data curation; formal analysis; investigation; methodology. **Sylvie Peyroche:** Investigation; methodology. **Myriam Normand:** Data curation; formal analysis; methodology; software. **Yacine Bertache‐Djenadi:** Formal analysis; investigation. **Thierry Thomas:** Writing – review and editing. **Hubert Marotte:** Writing – review and editing. **laurence vico:** Conceptualization; methodology; validation; writing – review and editing. **Marie‐Helene Lafage‐Proust:** Methodology; writing – review and editing. **Marthe Rousseau:** Conceptualization; funding acquisition; investigation; methodology; project administration; resources; supervision; writing – review and editing.

## Authors' roles

Study concept and design: MR, AVB, and LV. In vivo studies: MR, AVB, and NL. Micro‐CT analysis: DKN, NL, and YB. Bone histomorphometry: DKN, NL, and MHLP. Blood biomarker measurements: DKN, MTL, and SP. Gene expression analysis: DKN, MTL, and MT. Data analysis: DKN and MN. Data interpretation: DKN, MHLP, and MR. Data visualization and writing—original draft: DKN. Writing—review and editing: DKN, MHLP, and MR. Approving final version of manuscript: DKN, MHLP, TT, HM, LV, and MR.

5

### Peer review

The peer review history for this article is available at https://publons.com/publon/10.1002/jbm4.10655.

## Supporting information


**Supplemental Fig. S1.** OVX‐induced body weight variation in rats.

**Supplemental Fig. S2.** The morphological trabecular parameters in the proximal tibia in ex vivo cross‐sectional study.

**Supplemental Fig. S3.** Quantitative results of μCT ex vivo analysis in the distal metaphyseal femur expressed as BV/TV, Conn.D, Tb.N, Tb.Th, Tb.Sp, and SMI.

**Supplemental Fig. S4.** Location of skeletal sites was analyzed using μCT.

**Supplemental Fig. S5.** Changes in plasma PTH (*A*), serum calcium (*B*), plasma FGF23 (*C*), and serum phosphate (*D*) from baseline to follow‐up in the OVX and OVX Nacre groups.

**Appendix S1.** Supporting information.

**Supplemental Table S1.** Composition of Experimental Diets

**Supplemental Table S2.** The Supplemental Information Used in Bone Morphology Analysis by μCT

**Supplemental Table S3.** Primer Sequences Used in This Study

**Supplemental Table S4.** Quantification of μCT‐Derived Cortical Bone Microarchitectural Parameters at the Tibial and Femoral Diaphysis in Ex Vivo Cross‐sectional Study
