# Supplementary figures and images for: Protective Effect on Bone of Nacre Supplementation in Ovariectomized Rats
Source: JBMR Plus. 2022 Jul 15;6(9):e10655. doi: 10.1002/jbm4.10655 (PMC9464996; doi:10.1002/jbm4.10655)

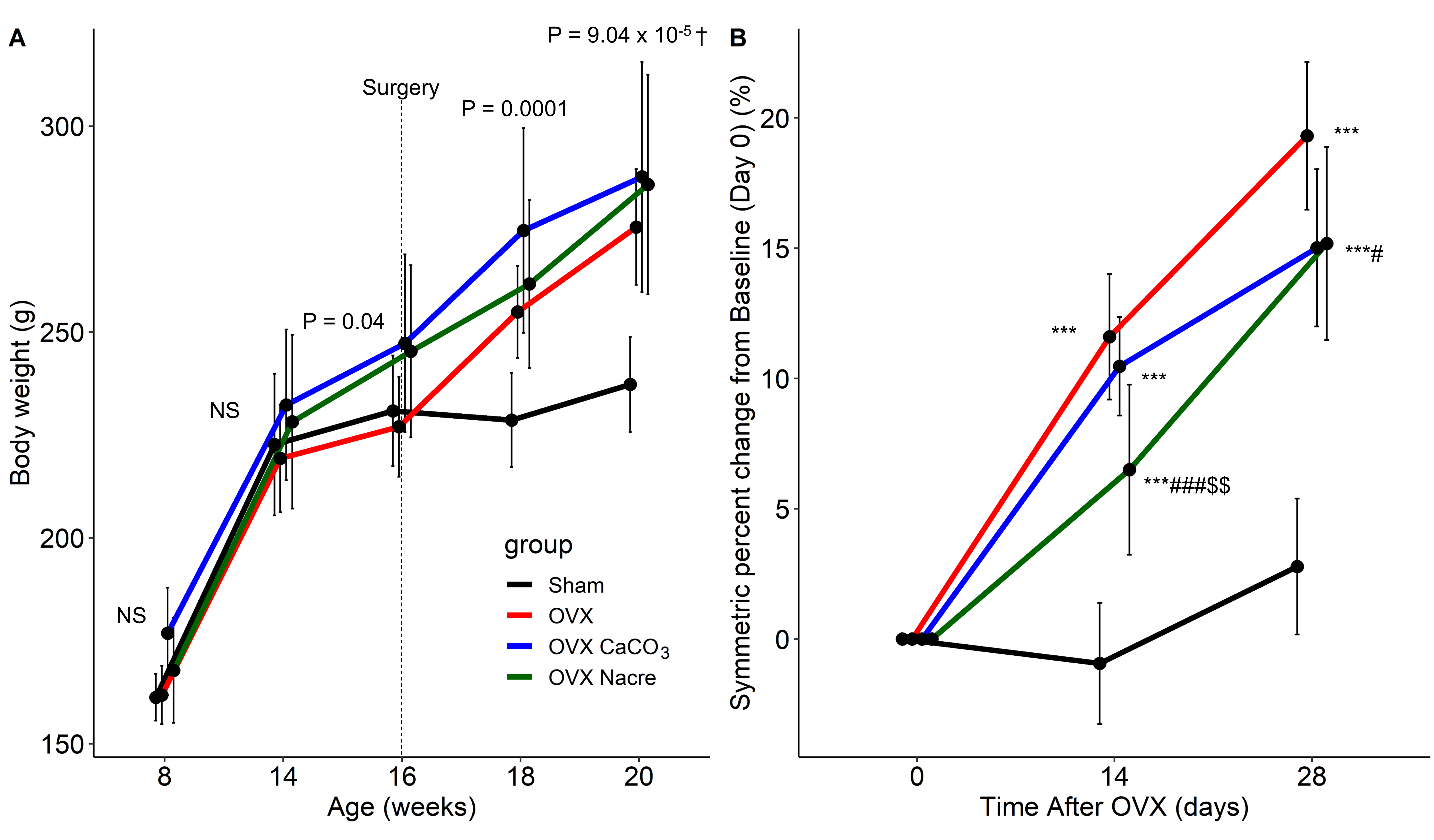

Supplement: Supplementary file 1 — Supplemental Fig. S1. OVX‐induced body weight variation in rats. [file JBM4-6-e10655-s007.tif]

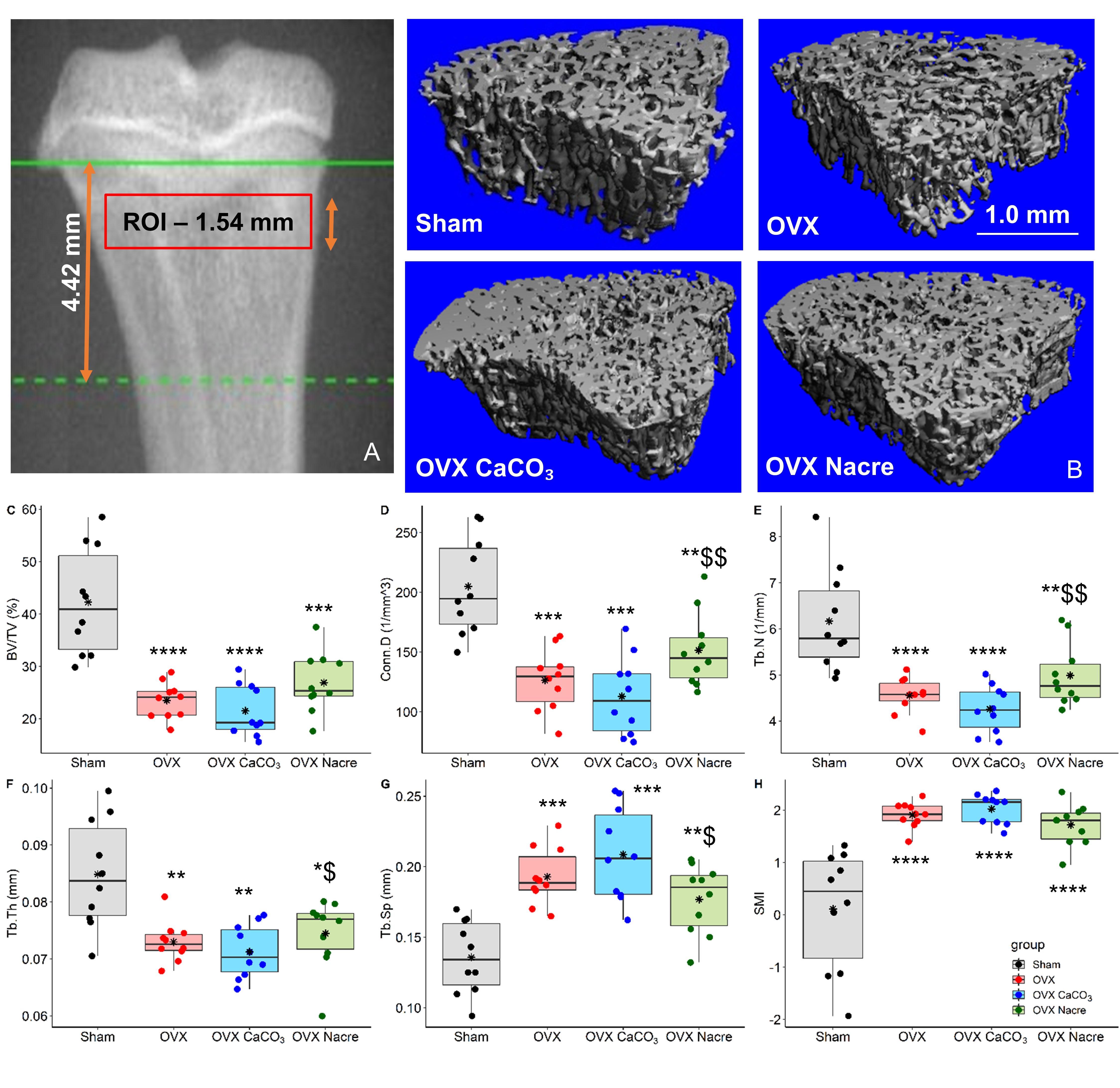

Supplement: Supplementary file 2 — Supplemental Fig. S2. The morphological trabecular parameters in the proximal tibia in ex vivo cross‐sectional study. [file JBM4-6-e10655-s002.tif]

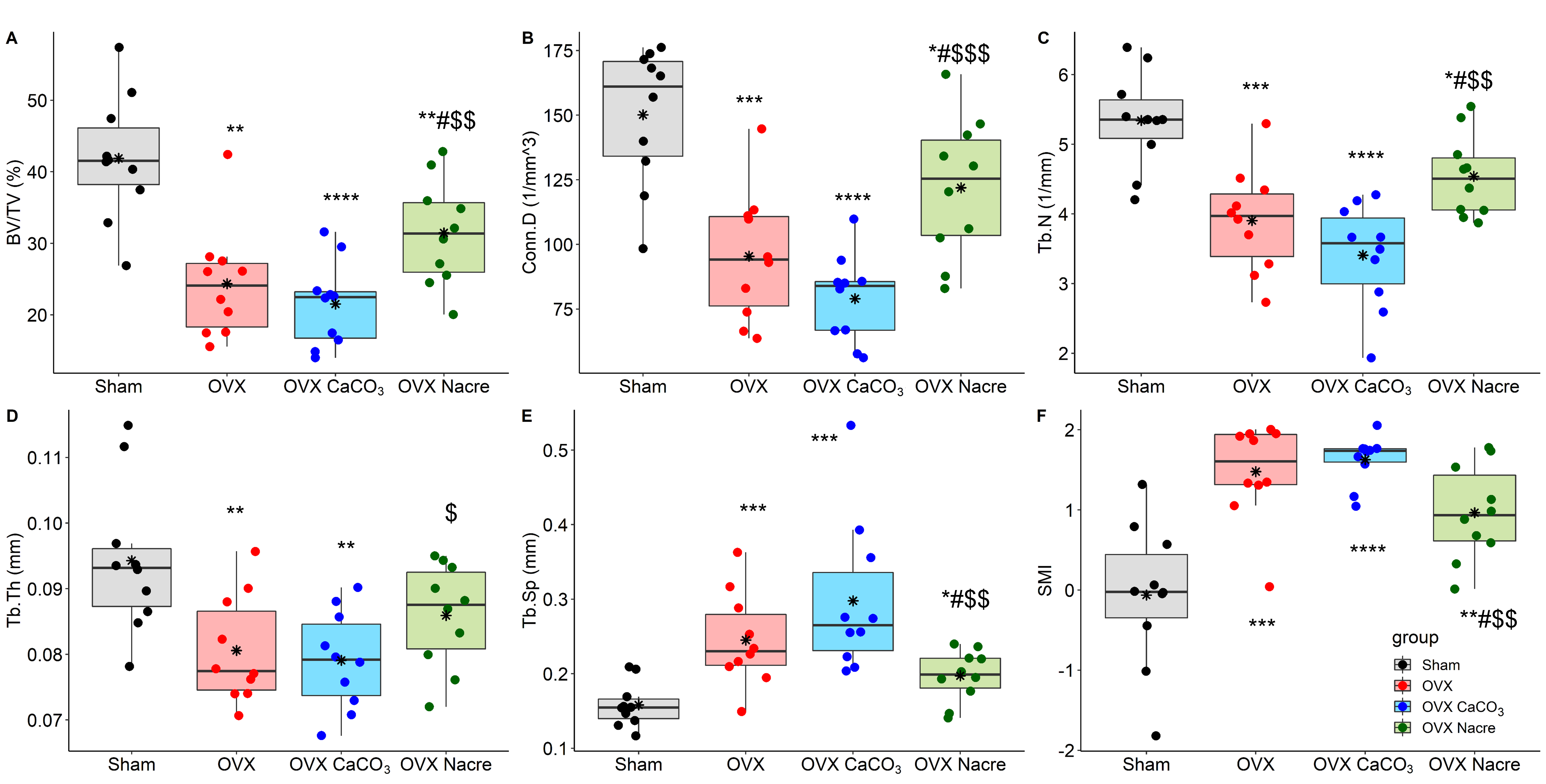

Supplement: Supplementary file 3 — Supplemental Fig. S3. Quantitative results of μCT ex vivo analysis in the distal metaphyseal femur expressed as BV/TV, Conn.D, Tb.N, Tb.Th, Tb.Sp, and SMI. [file JBM4-6-e10655-s001.tif]

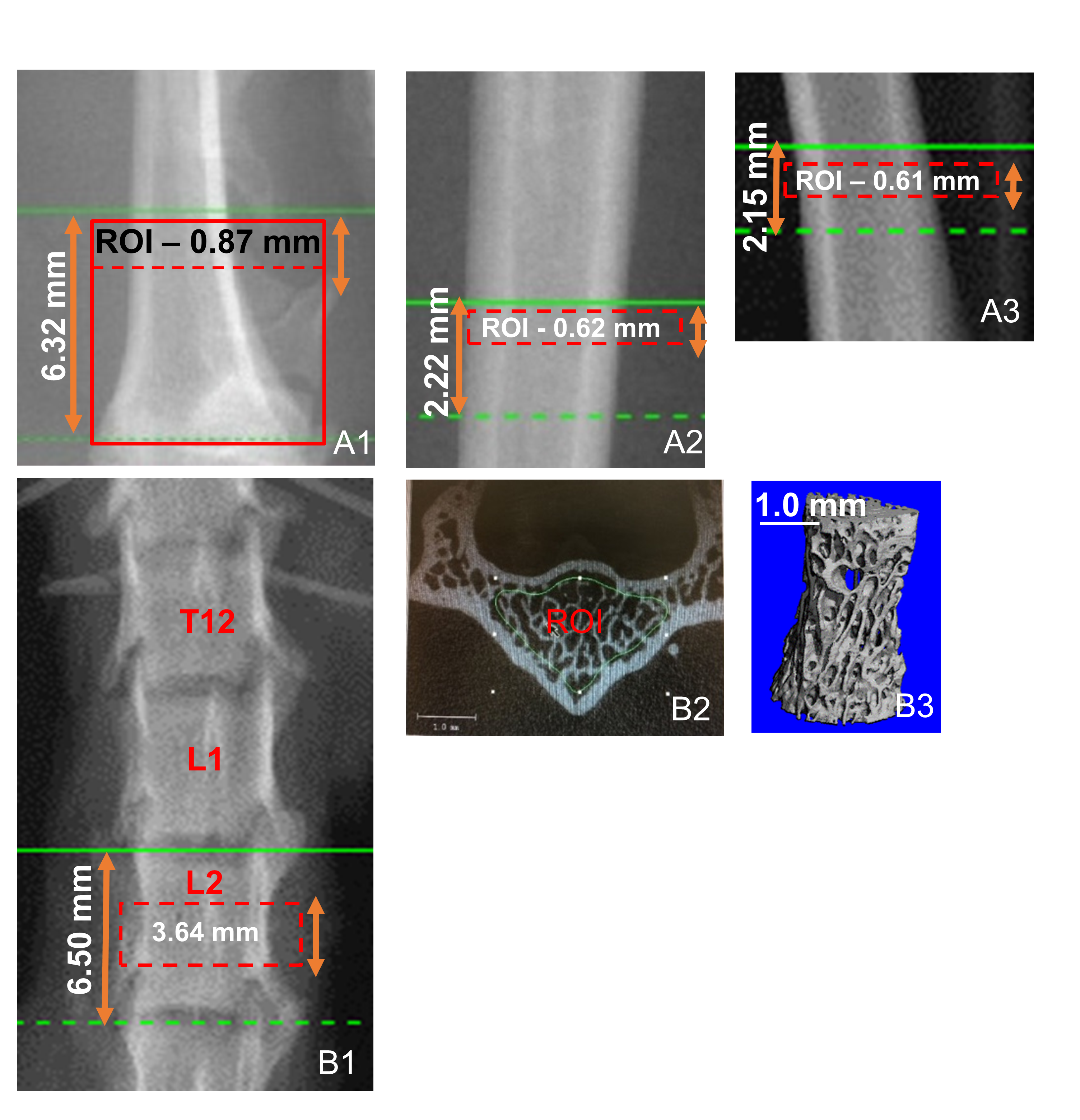

Supplement: Supplementary file 4 — Supplemental Fig. S4. Location of skeletal sites was analyzed using μCT. [file JBM4-6-e10655-s006.tif]

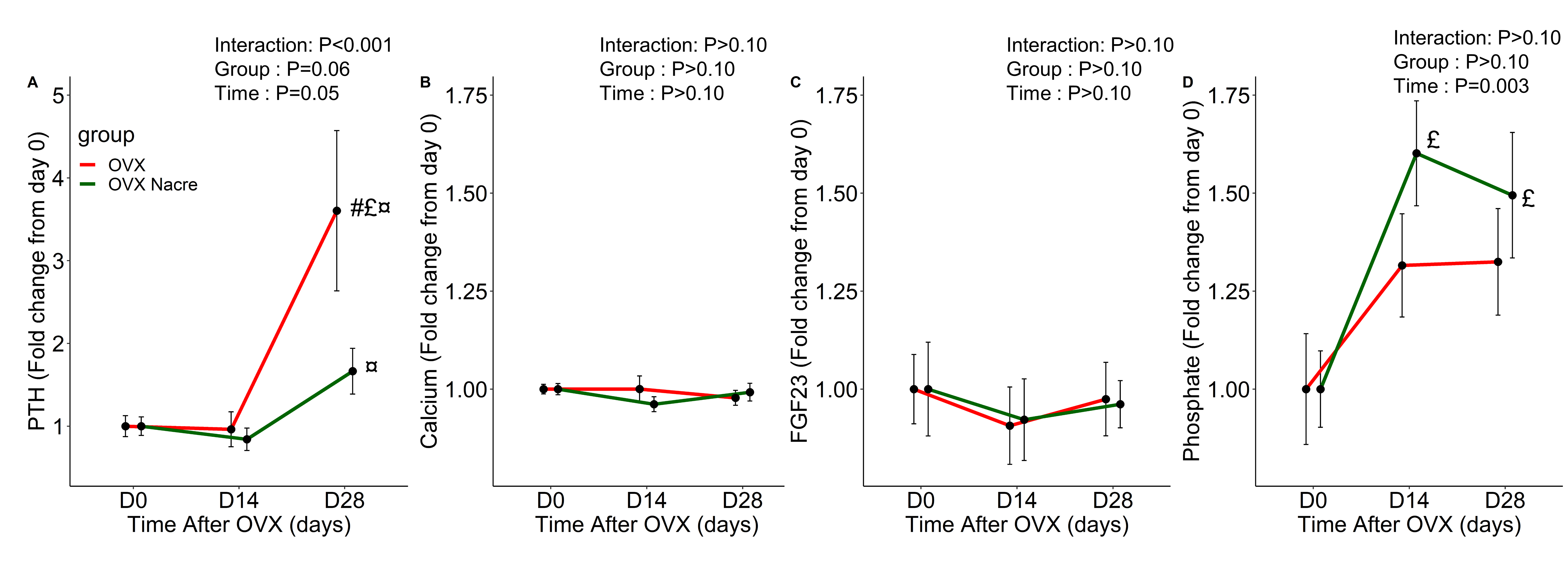

Supplement: Supplementary file 5 — Supplemental Fig. S5. Changes in plasma PTH (A), serum calcium (B), plasma FGF23 (C), and serum phosphate (D) from baseline to follow‐up in the OVX and OVX Nacre groups. [file JBM4-6-e10655-s010.tif]
