# Supplementary material for: Protective Effect on Bone of Nacre Supplementation in Ovariectomized Rats
Source: JBMR Plus. 2022 Jul 15;6(9):e10655. doi: 10.1002/jbm4.10655 (PMC9464996; doi:10.1002/jbm4.10655)
Supplement: Supplementary file 6 — Appendix S1. Supporting information. [file JBM4-6-e10655-s005.pdf]

Supplemental Materials

Protective effect on bone of nacre supplementation in ovariectomized rats

Dung Kim NGUYEN [1], Norbert LAROCHE [1], Arnaud VANDEN-BOSSCHE [1], Marie-Thérèse LINOSSIER [1], Mireille THOMAS [1], Sylvie PEYROCHE [1], Myriam NORMAND [1], Yacine BERTACHE-DJENADI [1], Thierry THOMAS [1,2], Hubert MAROTTE [1,2], Laurence VICO [1], Marie-Hélène LAFAGE-PROUST [1,2], Marthe ROUSSEAU [1,3]

Section 1: Microarchitectural analysis description by using Micro-computed tomography (μCT)

Section 2: Bone histomorphometry measurements

Section 3: Supplemental tables

Section 4: Supplemental figure legends and figures

Section 5: Sample ARRIVE guidelines table

Section 1: **Microarchitectural analysis description by using μCT**.

For *in vivo* μCT evaluation, cortical dimensions were determined on the last 58 slices (0.87mm) to obtain cross-sectional images for the volume of interest (VOI) drawn to exclude trabecular elements. Cortical analyses were measured at a threshold of 260 mg/cm^3, a Gaussian filter (sigma = 0.8, support = 1). Trabecular bone measurements at the proximal tibiae were made on 147 slices (2.21mm) away from the growth plate, to avoid the primary spongiosa. Trabecular bone analyses were performed on the contours of the cross-sectional images drawn to exclude cortical bone and were measured at a threshold of 190mg/cm^3, a Gaussian filter (sigma=1 and support=2). Both metric and non-metric bone parameters were evaluated. Metric parameters included: (i) trabecular bone morphometry: bone volume fraction (BV/TV, %); trabecular number (Tb.N, 1/mm); trabecular separation (Tb.Sp, mm);

trabecular thickness (Tb.Th, mm); connectivity density (Conn.D, 1/mm^3); (ii) cortical bone morphometry: cortical bone area (Ct.Ar, mm^2); cortical thickness (Ct.Th, mm); marrow area (Ma.Ar, mm^2), cortical porosity (Ct.Po, %). Non-metric parameters included the Structure Model Index (SMI), where an ideal value of 0 indicates the predominance of plate-like structure, while a value of 3 indicates the predominance of rod-like structure. Nomenclature conforms to recommendations of the American Society for Bone and Mineral Research [(1)].

For *ex vivo* µCT analyses of different skeletal sites. All bone tissues (tibiae, femora, lumbar spine) were placed in a tube filled with alcohol, blocked by the cotton plug, and scanned. Scan settings were as follows: isotropic voxel size 10.5 μm^3, 70kVp, 114µA, 1000 projections by 180 degrees with 250 ms integration time. The information on the scanning system and the VOI's dimension in detail were indicated in Tab. S2. Figures 2A and S4 were given to illustrate the scout-view, drawn ROI, and representative 3D images of VOI.

The second lumbar spine (LS2) was scanned between 2 discs to obtain 619 slices (6.50 mm). Trabecular parameters in the spine were determined using 347 slices (3.64 mm) centered at the body of LS2. Trabecular bone analyses were performed on contours of cross-sectional images, drawn to exclude cortical bone, as described for femoral trabecular bone.

Section 2: Bone histomorphometry measurements

After µCT scanning, the right tibiae were trimmed of soft tissue and fixed at 4°C with 70% ethanol for 14 days. The proximal one-third of the tibiae were cut, then embedded undecalcified in methyl methacrylate (MMA) following dehydration in 100% ethanol. Because of technical reasons, we lost 1 sample in each Sham and OVX $CaCO_3$ group. Thus, we performed these histomorphometric measurements with sample sizes as n=9/ Sham o**r** VX $CaCO_3$ group, n=10/ OVX, or OVX Nacre group. Nine µm-thick frontal sections for staining and 12 µm - thick sections for dynamic analysis were cut using a microtome (SM2500; Leica Biosystems Nussloch GmbH, Germany) and mounted on slides. The 9 µm – thick sections

were stained with Goldner's trichrome and tartrate-resistant acid phosphatase (TRAcP) to assess the microstructure and visualize osteoclasts, respectively. Unstained 9 μm – thick sections were analyzed using a fluorescence microscope (Zeiss Axio Scope A1, Germany) to determine the dynamic indices of bone formation based on tetracyclinlabelingng. Structural and dynamic histomorphometry indices were measured in the trabecular bone of the secondary spongiosa, using a semi-automatic image analysis system (Sony DXC 950P Camera; DMRB microscope, Leica Microsystems, Germany; Explora Nova software version 3.50, La Rochelle, France) at a magnification x 5 for microstructural indices and x 20 for cellular and dynamic indices. The primary static indices included bone volume fraction (BV/TV, %), trabecular thickness (Tb.Th, μm), trabecular number (Tb.N, 1/mm), trabecular separation (Tb.Sp, μm), Osteoclast Number per Bone Area (N.Oc/B.Ar, mm^2), Osteoclast Number per Bone perimeter (N.Oc/B.Pm, c/mm), Osteoclast Surface per Bone Surface (Oc.S/BS, %), Osteoclast Length (Oc.Le, μm). The dynamic parameters obtained from the tetracycline marker included single-labeled surface per bone surface (sLS/BS, %), and double-labeled surface per bone surface (dLS/BS, %). Mineral Apposition Rate (MAR, μm/day) was calculated from the mean distance between the labels divided by the number of days between labelings. Mineralizing Surface per Bone Surface (MS/BS, %) was calculated as ½ sLS/BS + dLS/BS. Bone - Formation Rate per Bone Surface (BFR/BS, $\mu m^3/\mu m^2$/day) was calculated by multiplying MS/BS by MAR. The histomorphometry nomenclature used in the present study was from a report from the American Society for Bone and Mineral Research Histomorphometry Nomenclature Committee [(2)].

Section 3: Supplemental tables.

Table S1. Composition of experimental diets.

Composition	Standard	OVX		
		**Standard**	**$CaCO_3$ supplement**	**Nacre supplement**
	**Sham †**	**OVX †**	**OVX $CaCO_3$ ‡**	**OVX Nacre ‡**
Acid amin Mix (g/kg) [a]	21.5	21.5	21.5	>21.5*
Fat acid Mix (g/kg) [b]	19.8	19.8	19.8	>19.8*
Vitamin Mix (g/kg) [c]	1.72	1.72	1.72	1.72
Soya trace	free	free	free	free
Mineral Mix (g/kg) [d]	27.84	27.84	28.84	28.79
Calcium included	8.5	8.5	9.5	9.45

Note: † Sham and OVX groups = standard diet, ‡ OVX $CaCO_3$ and OVX Nacre groups = standard diet supplemented with 0.25% $CaCO_3$ or nacre powder, respectively.

**Pinctata maxima*'s nacre contains 2.7% organic matrix (Bourrat X et al., *CrysEngComm*, 2007 [(3)]) ; 0.068 g nacre organic content per 1kg food.

[a] acid amin Mix – Arginine : 6.5, Cysteine : 2, Lysine : 4.4, Methionine : 1.8, Tryptophane : 1.5, Glycine : 5.3

[b] Fat acid Mix – Palmitic acid : 2.2, Stearic acid : 0.4, Oleic acid : 6, Linoleic acid : 11.2, Linolenic acid : 0.4

[c] Mineral Mix – Phosphate : 5, Sodium : 2.2, Potassium : 6.3, Magnesium : 1.9, Maganese : 0.09, Fer : 0.27, Copper : 0.016, Zinc : 0.060, Chlorure : 3.5

[d] Vitamine Mix – Vit A : 5, Vit D3 : 9, Vit B1 : 0.005, vit B2 : 0.006, Vit B5 : 0.01, vit B6 : 0.002, vit B12 : 2.10^{-5}, vit E : 0.025, vit K3 : 0.0025, Niacine : 0.07, acid Folic : 0.0005, Biotine : 0.00004, Choline : 1.6

Section 4: Supplemental figure legends

Figure S1. **OVX-induced body weight variation in rats**. The rat growth (weight, g) before and after the ovariectomized surgery (occurred at week 16 – Day 0) **(A)**. Data represent mean ± standard error (n=10 per group). † P-values were derived from a one-way ANOVA test. The body weight changes from baseline (Day 0) during 28 days of study **(B)**. The symmetric percent changes were calculated by finding the individual difference of values at baseline (*x0*) and follow-ups (D14 or D28) (*x1*), then divided this difference by the mean of baseline and follow-up values, i.e *(x1-x0)/mean (x0, x1) x 100.* Data represent mean ± standard error (n=10 per group). *** $P \leq 0.001$ *vs.* Sham; $^{\#}$ $P \leq 0.05$, $^{\#\#\#}$ $P \leq 0.001$ *vs*. OVX, $^{\$\$}$ $P \leq 0.01$ *vs*. OVX $CaCO_3$ (one-way ANOVA test followed by Tukey's HSD post hoc test to adjust for multiple comparisons).

Figure S2. **The morphological trabecular parameters in the proximal tibia in *ex vivo* cross-sectional study**. µCT scout image in which the box depicts a scanned region of 147 consecutive slices (10.5 µm/slice) analyzed for trabecular bone volume, as detailed in Methods (**A**). Representative 3D images of the trabecular bone microarchitecture in the right proximal tibia **(B)**. Quantitative results of µCT analysis expressed as BV/TV, Conn.D, Tb.N, Tb.Th, Tb.Sp, SMI (**C-H)**. Data represent as boxplots, and show all data points, with interquartile range (IQR) (height of the box), median (internal horizontal bar) (n=10 per group), and mean marked by a black asterisk in the box. * $P \leq 0.05$, ** $P \leq 0.01$, *** $P \leq 0.001$, **** $P \leq 0.0001$; * *vs.* Sham, $^{\#}$ *vs.* OVX, $^{\$}$ *vs.* OVX $CaCO_3$ (Kruskal-Wallis test followed by Mann-Whitney-Wilcoxon unpaired test with Benjamini-Hochberg adjustment).

Figure S3. **Quantitative results of µCT *ex vivo* analysis in the distal metaphyseal femur expressed as BV/TV, Conn.D, Tb.N, Tb.Th and Tb.Sp, SMI**. Data represent as boxplots, and show all data points, with interquartile range (IQR) (height of the box), median (internal horizontal bar) (n=10 per group), mean (black asterisk in the box, *). * $P \leq 0.05$, ** $P \leq 0.01$,

*** $P \leq 0.001$, **** $P \leq 0.0001$; * *vs.* Sham group, $^{\#}$ *vs.* OVX group, $^{\$}$ *vs.* OVX $CaCO_3$ group (Kruskal-Wallis test followed by Mann-Whitney-Wilcoxon unpaired test with Benjamini-Hochberg adjustment).

Figure S4. **Location of skeletal sites was analyzed using µCT**. µCT scout images of the appendicular bones: diaphyseal tibia in a longitudinal study (*in vivo*) **(A1),** and is a cross-sectional study (*ex vivo) (***A2)**, diaphyseal femur **(A3)** show the scanned region and ROIs for cortical bone measurements. µCT scout image of the axial bone, 2nd lumbar spine (LS2) shows the scanned region and trabecular ROI for trabecular bone measurement **(B1)**, 2-dimensional coronal slice shows ROI (drawn in green) through trabecular body region **(B2)**, representative 3-dimensional images of the trabecular bone microarchitecture **(B3)**.

Figure S5. Changes in plasma PTH (A) and serum Calcium (B), plasma FGF23 (C), and serum Phosphate (D) from baseline to follow-ups in the OVX and OVX Nacre groups. The data were represented as the mean ± standard error (vertical bar) (n=6 per group). P-values indicated the effect of group (between-subjects factor) and/or time (within-subjects factor) and the group x time interaction using two-way repeated-measures ANOVA, followed by Tukey's HSD posthoc test to adjust for multiple comparisons. $^{\#}$ $P \leq 0.05$. $^{\#}$ *vs.* OVX, $^{\$}$ *vs.* OVX $CaCO_3$; $^{£}$ *vs.* baseline at day 0, $^{¤}$ *vs.* follow-up at day 14.

Section 5: ARRIVE guidelines Checklist B (see in file pdf)

References

1. Bouxsein ML, Boyd SK, Christiansen BA, Guldberg RE, Jepsen KJ, Müller R. Guidelines for assessment of bone microstructure in rodents using micro–computed tomography. Journal of bone and mineral research. 2010;25(7):1468-86.
2. Dempster DW, Compston JE, Drezner MK, Glorieux FH, Kanis JA, Malluche H, et al. Standardized nomenclature, symbols, and units for bone histomorphometry: a 2012 update of the report of the ASBMR Histomorphometry Nomenclature Committee. Journal of bone and mineral research: the official journal of the American Society for Bone and Mineral Research. 2013;28(1):2.
3. Bourrat X, Francke L, Lopez E, Rousseau M, Stempflé P, Angellier M, et al. Nacre biocrystal thermal behaviour. CrystEngComm. 2007;9(12):1205-8.
