# Supplementary material for: Protective Effect on Bone of Nacre Supplementation in Ovariectomized Rats
Source: JBMR Plus. 2022 Jul 15;6(9):e10655. doi: 10.1002/jbm4.10655 (PMC9464996; doi:10.1002/jbm4.10655)
Supplement: Supplementary file 7 — Supplemental Table S1. Composition of Experimental Diets [file JBM4-6-e10655-s009.docx]

| **Table S1. Composition of experimental diets.** | | | | |
| --- | --- | --- | --- | --- |
| **Composition** | **Standard** | **OVX** | | |
|  |  | **Standard** | **CaCO_3_ supplement** | **Nacre supplement** |
|  | **Sham †** | **OVX †** | **OVX CaCO_3_ ‡** | **OVX Nacre ‡** |
| Acid amin Mix (g/kg) ^a^ | 21.5 | 21.5 | 21.5 | >21.5* |
| Fat acid Mix (g/kg) ^b^ | 19.8 | 19.8 | 19.8 | >19.8* |
| Vitamin Mix (g/kg) ^c^ | 1.72 | 1.72 | 1.72 | 1.72 |
| Soya trace | free | free | free | free |
| Mineral Mix (g/kg) ^d^ | 27.84 | 27.84 | 28.84 | 28.79 |
| Calcium included | 8.5 | 8.5 | 9.5 | 9.45 |
| Note: † Sham and OVX groups = standard diet, ‡ OVX CaCO_3_ and OVX Nacre groups = standard diet supplemented with 0.25% CaCO_3_ or nacre powder, respectively.  **Pinctata maxima*’s nacre contains 2.7% organic matrix (Bourrat X et al., *CrysEngComm*, 2007 ^(^[^3^](#_ENREF_3)^)^) ; 0.068 g nacre organic content per 1kg food.  ^a^ acid amin Mix – Arginine : 6.5, Cysteine : 2, Lysine : 4.4, Methionine : 1.8, Tryptophane : 1.5, Glycine : 5.3  ^b^ Fat acid Mix – Palmitic acid : 2.2, Stearic acid : 0.4, Oleic acid : 6, Linoleic acid : 11.2, Linolenic acid : 0.4  ^c^ Mineral Mix – Phosphate : 5, Sodium : 2.2, Potassium : 6.3, Magnesium : 1.9, Maganese : 0.09, Fer : 0.27, Copper : 0.016, Zinc : 0.060, Chlorure : 3.5  ^d^ Vitamine Mix – Vit A : 5, Vit D3 : 9, Vit B1 : 0.005, vit B2 : 0.006, Vit B5 : 0.01, vit B6 : 0.002, vit B12 : 2.10^-5^, vit E : 0.025, vit K3 : 0.0025, Niacine : 0.07, acid Folic : 0.0005, Biotine : 0.00004, Choline : 1.6 | | | | |
