# Supplementary material for: Protective Effect on Bone of Nacre Supplementation in Ovariectomized Rats
Source: JBMR Plus. 2022 Jul 15;6(9):e10655. doi: 10.1002/jbm4.10655 (PMC9464996; doi:10.1002/jbm4.10655)
Supplement: Supplementary file 8 — Supplemental Table S2. The Supplemental Information Used in Bone Morphology Analysis by μCT [file JBM4-6-e10655-s008.docx]

| **Table S2**. **The supplemental information used in bone morphology analysis by µCT.** | | | | | | | | |
| --- | --- | --- | --- | --- | --- | --- | --- | --- |
| **Bone site** | **Voxel size (µm^3^)** | **Gaussian filter** | | **Threshold (mg/cm^3^)** | **Dimension (mm)** | | **Type of study** | |
|  |  | **Sigma** | **Support** |  | **Acquisition** | **Evaluation** | ***In vivo*** | ***Ex vivo*** |
| Trabecular proximal tibia | 15 | 1 | 2 | 190 | 6.32 [421] | 2.21 [147] | x |  |
| Cortical proximal tibia | 15 | 0.8 | 1 | 260 | 6.32 [421] | 0.87 [58] | x |  |
| Trabecular proximal tibial metaphysis | 10.5 | 1 | 2 | 190 | 4.42 [421] | 1.54 [147] |  | x |
| Cortical tibial diaphysis | 10.5 | 0.8 | 1 | 260 | 2.22 [211] | 0.62 [59] |  | x |
| Trabecular distal femoral metaphysis | 10.5 | 1.5 | 2 | 290 | 6.63 [631] | 1.54 [147] |  | x |
| Cortical femoral diaphysis | 10.5 | 0.8 | 1 | 260 | 2.15 [205] | 0.91 [87] |  | x |
| Trabecular 2^nd^ lumbar spine | 10.5 | 1.2 | 2 | 240 | 6.50 [619] | 3.64 [347] |  | x |
| Note: The number of slices was indicated in square brackets []. | | | | | | | | |
