# Supplementary material for: Protective Effect on Bone of Nacre Supplementation in Ovariectomized Rats
Source: JBMR Plus. 2022 Jul 15;6(9):e10655. doi: 10.1002/jbm4.10655 (PMC9464996; doi:10.1002/jbm4.10655)
Supplement: Supplementary file 9 — Supplemental Table S3. Primer Sequences Used in This Study [file JBM4-6-e10655-s003.docx]

| **Table S3. Primer sequences used in this study** | | |
| --- | --- | --- |
| Genes (protein encoded) | **Sense (5’-3’)** | **Antisense (5’-3’)** |
| *Hprt1* | GTTGGATACAGGCCAGACTT | GCCACATCAACAGGACTCTT |
| *Runx2* (RUNX2) | CAGACCAGCAGCACTCCATA | CGCCAGACAGACTCATCCAT |
| *Bglap* (Osteocalcin) | CAACTCGGTGCAGACCTAGC | GAGGTAGCGCCGGAGTCTAT |
| *Spp1* (Osteopontin) | GGAGAAGGCGCATTACAGCA | CGTCATCGTCGTCGTCATCA |
| *Sparc* (Osteonectin) | AGGTGTGCAGCAATGACAAC | ATTCGGTCAGCTCAGAATCC |
| *Col1a2* (Collagen type 1 α2) | ATTGCGTACCTGGACGAGGA | GGCAGGCGAGATGGCTTATT |
| *Bmp2* (BMP-2) | TTGAGGCTGCTCAGCATGTT | CTCGATGGCTTCTTCGTGAT |
| *Nfatc1* (NFATc1) | CGTGGAGAAGCAGAGCACAG | CTTGCACAGGTCTCGGTCAG |
| *Acp5* (TRAP) | CAGCCAAGGAGGACTATGTT | ACACCGTTCTCATCCTGAAG |
| *Cathepsin K* (Cathepsin K) | AGTGCCACCTTCGCGTTCCT | TAGCCGCCTCCACAGCCATA |
| *Rank* (RANK) | ATCGTCCTGCTCCTCTTCAT | ACTTCTTGCTGGCTGGAGTT |
| *Rankl* (RANKL) | GACAGCACGCGCTGCTTCTA | CCACATCGAGCCACGAACCT |
