# Supplementary material for: Protective Effect on Bone of Nacre Supplementation in Ovariectomized Rats
Source: JBMR Plus. 2022 Jul 15;6(9):e10655. doi: 10.1002/jbm4.10655 (PMC9464996; doi:10.1002/jbm4.10655)
Supplement: Supplementary file 10 — Supplemental Table S4. Quantification of μCT‐Derived Cortical Bone Microarchitectural Parameters at the Tibial and Femoral Diaphysis in Ex Vivo Cross‐sectional Study [file JBM4-6-e10655-s004.docx]

| **Table S4. Quantification of µCT-derived cortical bone microarchitectural parameters at the tibial and femoral diaphysis in *ex vivo* cross-sectional study.** | | | | | | | | | | |
| --- | --- | --- | --- | --- | --- | --- | --- | --- | --- | --- |
| **Characteristic**  **(unit)** | **Tibial diaphysis** | | | |  | **Femoral diaphysis** | | | |  |
|  | **Group** | | | |  | **Group** | | | |  |
|  | **Sham** | **OVX** | **OVX CaCO₃** | **OVX Nacre** | **P-value†** | **Sham** | **OVX** | **OVX CaCO₃** | **OVX Nacre** | **P-value †** |
| Ct.Ar  (mm²) | 3.70  (3.64-3.73) | 3.97***  (3.82-4.05) | 4.04  (3.64-4.14) | 3.98**  (3.90-4.13) | **0.02** | 5.21  (4.96-5.34) | 5.60*  (5.52-5.65) | 5.50  (5.29-5.86) | 5.43*  (5.24-5.72) | 0.06 |
| Ct.Th  (mm) | 0.55  (0.53-0.56) | 0.56  (0.51-0.59) | 0.57  (0.55-0.59 | 0.57  (0.55-0.59) | >0.10 | 0.61  (0.60-0.62) | 0.62  (0.61-0.63) | 0.60  (0.59-0.62) | 0.60  (0.58-0.63) | >0.10 |
| Ma.Ar  (mm²) | 1.39  (1.26-1.48) | 1.41  (1.29-1.48) | 1.43  (1.29-1.55) | 1.51  (1.24-1.78) | >0.10 | 2.86  (2.57-3.90) | 2.96  (2.70-3.32) | 3.16  (2.61-3.38) | 2.96  (2.77-3.43) | >0.10 |
| Ct.Po  (%) | 2.91  (2.85-3.03) | 3.36  (3.10-3.58) | 3.12  (2.95-3.40) | 3.10  (3.06-3.35) | 0.06 | 1.30  (1.23-1.32) | 1.42  (1.29-1.48) | 1.44  (1.39-1.55) | 1.39*  (1.26-1.59) | >0.10 |
| Note: †Kruskal Wallis test  Post hoc pairwise comparisons were conducted using Mann-Whitney-Wilcoxon unpaired tests with the Benjamini-Hochberg adjustment.  P-values in bold indicated statistical significance at level of 5% (P ≤ 0.05). *P ≤ 0.05, **P ≤ 0.01, ***P ≤ 0.001; * *vs*. Sham, ^#^ *vs*. OVX.  Values are median (interquartile range), n=10 per group | | | | | | | | | | |
